# Novel subgroups of functional ability in older adults and their associations with adverse outcomes

**DOI:** 10.1186/s12877-022-03081-9

**Published:** 2022-05-04

**Authors:** Ying Han, Liangwen Zhang, Ya Fang

**Affiliations:** 1 State Key Laboratory of Molecular Vaccinology and Molecular Diagnostics, School of Public Health, Xiamen University, Xiamen, Fujian 361102 PR China; 2 School of Economics, Xiamen University, 422 Siming South Road, Xiamen, Fujian 361005 PR China

**Keywords:** Disability, Latent class analysis, Alkire-Foster, Older adults, Mortality, Functional assessment

## Abstract

**Background:**

There is no general agreement on a standard form of functional classification in older adults and is mainly assessed by Activities of Daily Living (ADL) and/or Instrument Activity of Daily Living. A refined classification based on evaluation the limitations of intrinsic capacity, environment and social interaction, could provide a basis to predict the future disability and identify individuals with increased risk of adverse outcomes.

**Methods:**

A new functional classification among older adults aged 60 and over was conducted by latent class analysis and compared with the traditional classifications, based on the China Health and Retirement Longitudinal Study. To further investigate the scientific validity of this new classification, associations with 7-year mortality and ADLs impairments among categories were tested by using Survival curves and Cox proportional hazard models. This was followed by the confirmatory analysis related to the prospective data. Competing risk analysis was also performed to analysis the sensitivity to further support our conclusions.

**Results:**

Five categories were identified among 5,992 older adults which gave the best fitting, yielding a significant Bootstrap Likelihood Ratio Test (*p* < 0.001) and Lo-Mendell-Rubin adjusted likelihood ratio test (*p* < 0.001), with an entropy over 0.80. The presence of five categories: “health” (34.0%), “sub-disorder status” (36.6%), “acute diseases” (10.3%), “somatic functional disorder” (7.7%), and “viability disorder” (11.4%), which matched well with the functional independence rates by the international classifications. Among them, those in “sub-disorder status” were considered as an intermediate status between disability and health. The findings also revealed that those who were in “acute disease”, “somatic functional disorders”, “health” and “sub-disorder status” had a significant lower risk of mortality and ADLs limitations than “viability disorder”. And the risks gradually increased towards the less functionally independent end of the classification. However, the distribution of characteristics among five categories were in a synchronous change, indicating a stable classification.

**Conclusions:**

A new classification representing the functional heterogeneity of older adults could effectively stratify the risk of mortality and ADLs limitations. Identifying the clusters of functional decline might be useful in predicting subsequent ageing trends, designing personalized intervention, and delaying the progression of disability and preventing its occurrence.

**Supplementary Information:**

The online version contains supplementary material available at 10.1186/s12877-022-03081-9.

## Background

According to the recent Decade of Healthy Ageing Baseline Report of the World Health Organization (WHO), functional ability includes individuals’ physical and mental abilities, the environment where people live, and the manner people interact within that [1]. Maintaining functional independence is essential for older adults to keep healthy and to delay or prevent the possible adverse outcomes in ageing process [2]. However, the WHO’s report highlighted the fact that in 2021, over 142 million of older people were unable to meet all of their basic daily needs, which accounts for 14% of all people aged 60 and above, all over the world [1]. As people age, their health needs tend to become increasingly chronic and complex. Therefore, it is urgent to assess the level of function in older adults, as well as valuable to develop responses to the problem of population ageing for health policy makers.

The diverse needs of older adults should be regarded as a continuum of functions, covering functional independence from highest to lowest [3]. The targeted interventions can be developed for different functional levels to maintain or improve functional independence and prevent adverse health outcomes [4, 5]. Functional capacity should be measured by using an instrument that assesses individuals’ intrinsic abilities, the environment where people live and their interactions within it [1, 6]. From a holistic life course perspective, intrinsic ability refers to the combination of all the physical and mental abilities of individuals that is also regarded as the physiological basis for healthy ageing [7]. Scholars usually assessed intrinsic abilities indirectly by measuring basic activities of daily living (BADL) and/or instrumental activities of daily living (IADL). BADL is important to sustain self-care (e.g., dressing, eating), while IADL are critical for independent living (e.g., shopping, preparing a meal). However, this approach is only used to identify people with severe loss of function, which approaches to disability status [8–10]. On this basis, a disability process model (VJ94) was proposed, and defined disability as the inability to perform normal activities in social and economic life [11]. Those activities are not limited to the ADLs, IADLs, etc. [12]. The VJ94 model is a major refinement over the medical model of disability that elucidates the role of the underlying physical, emotional, and social contexts in which pathology develops into functional decline (impairments of comprehensive functions), that is followed by disability [13]. The studies investigating levels of functioning in relation to survival or adverse outcomes have focused on the end of the continuum (intrinsic capacity impairment) or early stages (limitations of environment and social interaction), but few have comprehensively analyzed the full continuum from health to severe disability [12, 14, 15]. However, it is imperative to look into a wider range of functional continuum [16], because it is of great value and practically significant to take appropriate intervention measures for those who are at risk of adverse outcomes or functional decline before the disability state occurs.

The influence of environment and interactions within it on enabling ageing well should be taken into account while classifying groups on functional ability. The environment which is referred as a place where people live, that includes the home, community, society, and all the elements within them that contribute to shape the behavior and characteristics of older adults at certain levels of intrinsic competence. In agreement with a framework of Capability approach [17], there are factors that allow individuals to convert resources into capabilities. Those conversion factors could be internal and external. The internal conversion factors refer to physical conditions. The external conversion factors included environmental characteristics [18, 19]. An environment could provide a range of resources or barriers that are essential factors in enabling older adults to adapt or compensate for their losses of intrinsic abilities [20]. Thus, the development of a favorable and age-friendly environment is an effective way to optimize functional capacity and develop healthy ageing [21]. However, few studies to date have incorporated the environment in the assessment process of the functional continuum, which means that there may be a large number of older adults who are unable to meet their basic needs, but remained unidentified. In addition, these studies do not capture a person’s real-time functional ability in a dynamic way, taking into account a person’s intrinsic capacity and interaction with their environment, which including activities in public spaces, participation in educational and physical activity opportunities and ways to contribute to society [22–24]. Therefore, based on clarifying the optimized contents within each domain of functional ability, we include intrinsic capacity, environment, social interaction, and a pre-disability state in the functional continuum construct, to fill in the gap of small losses of functioning and standardize the evaluation and classification of function in population-based surveys. As well the use of the capability approach is rather an innovative way of assessing disabling situations.

The national guidelines maintain the information on the functional classification, but the classification has not been updated much in the past 10 years, and very few attempts have been made to explore the functional heterogeneity in terms of resources and society [25, 26]. We searched Web of Science and Google research up to Jan 1,2019, using the Medical Subject Heading terms “disability” “functional assessment”, and “classification”. We identified several calls from expert groups for revision of the classification, but few efforts have made to include intrinsic capacity, environment and social interaction, and implemented in the home-based community.

Existing Long-term care insurance is restricted by the fact they cover severe disability when it has developed, but there is no way to predict or care for which older population will become functional impairments and need intensified treatment or prevention measures [27]. Evidence suggests that early intervention is essential to prevent life-shortening complications caused by functional decline, as the ensuing severe disability appears to exacerbate the mechanism of apoptotic and is irreversible [28, 29].

A refined classification could provide a powerful tool to identify those functional decline and most likely to experience severely disabled at an early stage. It also could be a better predictor of adverse events in older adults, more predictive than multimorbidity or polypharmacy, and that it should be considered an assistance for clinicians to optimal treatment [30, 31]. To this end, latent class analysis (LCA) was used to classify functional ability. There are two main reasons for us to use it. First, LCA is a method of classifying heterogeneous samples by probability distribution, i.e., the presence of a latent category variable that can classify a heterogeneous whole into multiple subgroups and can be viewed as a special case of model-based clustering, for multivariate discrete data. LCA is applicable to our research, for these functional indicators are multivariate discrete data [32]. Second, LCA is increasingly used to study population heterogeneity in health and support decision-making. It has been applied to typify or cluster clinical patients with severe illness, depression, and medical conditions to support the development of treatments or prevention programs, with high application value and reliability [33, 34].

Therefore, an LCA was used to classify the older population combining intrinsic capacity, environment and society interaction in this study, to better capture the full stage of it. This new functional classification will be studied in relation to mortality and ADLs impairments, to see whether it is effective in stratifying the risk of them. The main objective was to investigate whether the new classification is validated and effective, and whether it is associated with mortality and ADLs impairments in older adults during 7-year follow up, using data from the China Health and Retirement Longitudinal Study (CHARLS), a population-based cohort study of China.

## Methods

### Theoretical framework

Based on the framework “capability approach” theory elaborated by Amartya Sen, functional ability is closely related to the accessibility of resources and society, as well as the individual impairment [35]. In agreement with WHO framework for healthy ageing, we have included intrinsic capacity, environment and social interaction in the functional continuum construct. To this end, this study classified functional ability according to the multidimensional indexes constructed in previous studies.

### Study population

We used data from the first wave of the CHARLS (2011) and follow-up data on mortality over 7 years (2011–2018). The CHARLS is a population-based cohort study among adults aged 45 and older from 28 provinces and municipalities of China. Details on the methods and sampling have been published previously [36]. Within a total sample size of 17,708 in the 2011 baseline data, groups such as missing variables, incomplete information, and institutional pensions were excluded, and then a sample of 5992 older people aged 60 and above living at the home-based community were selected and included in the study. Of the 3859 people who had independent ADLs were also included in the current analysis. Based on data availability, relevant indicators and dimensions from systematic literature analysis, our conceptual framework of function of older people was defined in terms of three aspects of intrinsic capacity, environment and social interaction [37, 38]. And all the data information used in analysis mainly covers issues on sociodemographic characteristics, family, behavior, cognitive, psychological, environmental and biological factors that affect health and longevity. The present research protocol was approved by the Biomedical Ethics Review Committee of Peking University (IRB00001052–11,015). Ethics approval for the use of the CHARLS data was obtained from the University of Newcastle Human Research Ethics Committee (H-2015–0290). All methods were conducted in accordance with the principle of the Declaration of Helsinki, and all the participants or their legal representatives gave their written informed consent before any study procedures began.

1. Sociodemographic characteristics: age, sex, place of residence (urban and rural), educational level
2. Household factors: marital status, per capita annual household income
3. Biological factors: blood pressure, Body Mass Index (BMI)
4. Daily behavior: social activities, motor ability
5. Health behavior: sleep duration, self-rated health, life satisfaction, pain, formal (paid) and informal (unpaid) care
6. Multidimensional functional ability related variables

### Exclusion for data analysis

**Fig. 1:**
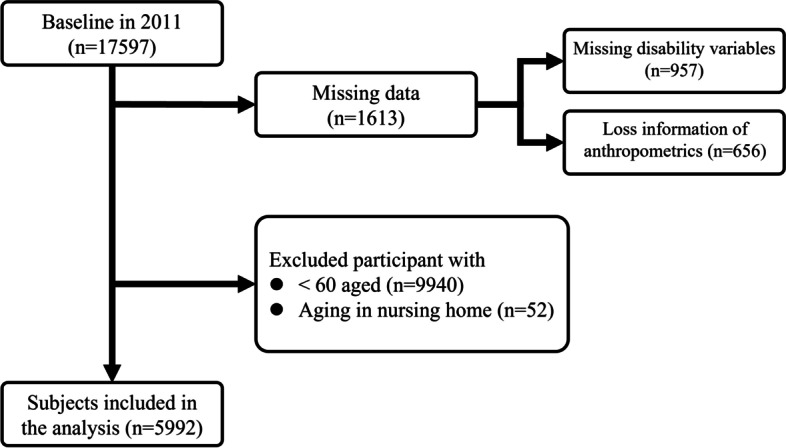
Baseline study population flow chart. Note. A total of 17,597 older people was screened. A total of 5992 older people were included who meet our study criteria in baseline

**Fig. 2:**
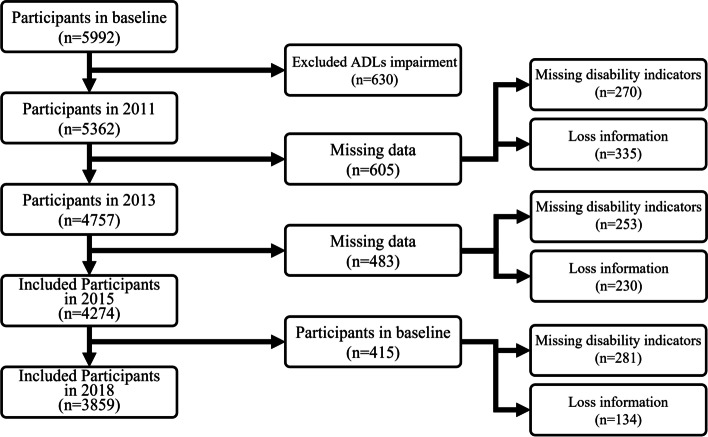
Participants flow chart. Note. Participants who are suffering from ADLs impairments in baseline, and losing important information were excluded. A total of 3859 older adults were included to assess the association of the new functional classification with 7-year all-cause mortality and upcoming ADLs impairments

After excluding the missing of functional indicators, losing information, and participants who did not meet our study criteria, a total of 5992 older adults were included in this analysis. To assess the association of the new functional classification with 7-year all-cause mortality and ADLs impairment, after excluding those data missing, there were 3859 participants in 2018 (Figs. 1 and 2).

### Variables

Participants were categorized into five groups representing the functional continuum, based on 29 multidimensional functional indexes. There were three aspects (intrinsic capacity, environment and social interaction) in terms of those indexes. Intrinsic capacity includes self-care, motor ability, ability of processing diseases, cognitive mental status and communication skills, and medical conditions. For example, self-care impairment was defined as the presence of at least one negative index, which indicated reducing ability to perform ADL, IADL [39] and frailty [40]. Motor ability included balance, falls, postural transition, arm stretching and mobility, while this capability impairment was evaluated by failing to complete one of them. Older people who seek medical treatment, exceptional treatment or use assistive device, without adopt self-treatment were regarded as a loss in the ability of processing disease. Cognitive mental status and communication skills were experience-dependent, and encompasses cognition, memory, vision, hearing, sadness and depression condition, as this ability impairment was defined to exist with at least one decline capability. The measurement of medical condition was derived from medical records where chronic diseases and physical disability are registered.

The assessment of functional capacity in environments includes both care resources and home setting for older adults. Care resources constituted of access of caregivers and living arrangement. Older people who lived alone and far away from their children or lacking of caregivers were defined as sources of care deficiency. Home settings as an environmental characteristic could be converted into capabilities, while limitation of it was defined as none of the elevator in a four-story apartment and accessibility.

Social interactions impairment results in activity limitations like restriction in interacting with friends, joining community club and community-related organization, and failure to help friends. A summary statistic of the variables applied for this study was presented in Table S1 in supplement.

### Mortality

All deaths that occurred between the baseline measurement and the follow-up of CHARLS (2013–2018) were recorded. The occurrence of death was noted and checked with the death registration in the database (100% ascertainment for the current study sample).

### Covariates

The study controlled for other functional ability attributes that affect mental and physical health. These included age, gender, marital status, place of residence(rural or urban), household per capita income, body mass index (BMI), educational level and numbers of children in the family. A summary statistic of the variables applied for this study was presented in supplementary Table S1.

### Statistical analysis

Statistical data processing was performed using stata version 16.0. Based on the previously constructed dimensions and indexes, Latent class analysis (LCA) was performed to identify the latent classes of functional ability among older adults for the year 2011, 2013, 2015 in Mplus8.0. LCA models with 2–7 classes were performed to find the best fitting model. We reported the value of Akaike Information Criterion (AIC) and Bayesian information criterion (BIC) values for each model, where a lower score indicates a better fit and a higher entropy to evaluate the model fit [41]. If entropy is greater than 0.8, which means the accuracy of classification is more than 90%. Then, we used Bootstrap Likelihood Ratio Test (BLRT) and Lo-Mendell-Rubin adjusted (LMR-A) test to compare n-class model with n-1-class models, with smaller classes indicating more optimal. In addition to the model-fitting metrics, the simplicity and interpretability of the model were also considered. We also tested the measurement invariance of LCA *p* across sex, age and living region. The latent classes of functional ability for older adults were named according to the distinctive conditional probability of multidimensional indicators in each category [42].

Descriptive analysis was performed to present sociodemographic characteristics according to the five categories of the total samples. The univariate logistic regression model was used to examine the association between multidimensional functional indicators with mortality, and to further confirm the assigned values of 29 indicators (negative or positive). Bonferroni correction was used to determine significance for multiple tests.

To evaluate the association of the new functional classification with 7-year all-cause mortality and 7-year all-cause ADLs impairment, Cox proportional hazard models were fitted in R version 4.1.0. adjusted for age, sex, marital status, place of residence, household per capita income, BMI, educational level and numbers of children. Those who died or got ADLs impairment during follow-up were censored on the date of their death or impairment. Survivors were censored on the date of the last interview (approximately 7 years since the baseline). To demonstrate the differences in 7-year survival between the functional categories, survival curves were fitted in R version 4.1.0. Factor analysis was used to determine the percentage of mortality variability explained by the different variables of the study.

In addition, a competing risk analysis was performed by using the “cmprsk” R package to evaluate the risk of ADLs impairment from the year 2011 to 2013 for different functional categories, treating non-ADLs deaths, including, car accidents, accidents, natural disasters, poisonings, and injuries as competing events. This model was used as a sensitivity analysis to further support our conclusions.

## Results

### Classification of multi-dimensional functional ability and confirmatory analysis

#### Latent class analysis

A total of 5992 participants (49.97% men, 36.25% age over 70 years, 20.46% living in urban) were included in latent functional classes in 2011. The fitting indices of the different class models examined are shown in Table 1. The two-, three-, four- and five-class model yielded a significant LMR-A and BLRT result at *p* < 0.05. Both the six- and seven-class models yielded a significant LMR-A result at *p* < 0.05, but not a significant LMR-A result. The five-class model had both a significant of LMR-A and trustworthy BLRT result, with an entropy value higher than 0.8, and had the lowest AIC and BIC values among the five models considered. Thus, based on all of the fitting indices examined and on the interpretability of the symptom profiles of the classes (consistent with the study hypotheses), the five-class model was selected. In table S2, we compared the functional dependence rates measured by the internationally adopted classifications (ADL, IADL, and both ADL and IADL) with the results measured by the new classification in this study, and found that the new classification matched well with the functional decline rates. This indicates the new functional classification is reliable and accurate. The results also presented that the range of 58.39% to 76.22% of perfectly healthy or independent older adults measured by the traditional method is equivalent to the sum of the fourth and fifth categories of the new classification (70.65%), indicating that both the fourth and fifth categories belonged to the traditional health category. Together with the conditional probability distribution with reference to Table S3, we found that the fourth category had the best functional condition among all the categories, thus, the fourth category could be defined as “health”, which is similar to the WHO’s definition of health as “a state of complete physical, mental, and social well-being and not merely the absence of disease or infirmity”.

**Table 1:** Latent class model and fit indices

Model	K	AIC	BIC	SSA-BIC	Entropy	LMR-A *p*-value	BLRT *p*-value	Classification probability
1	42	207,786.964	208,068.29	207,934.823				1
2	85	196,672.913	197,242.259	196,972.152	0.812	< 0.001	< 0.001	.62/.38
3	128	194,977.932	195,835.3	195,428.551	0.862	< 0.001	< 0.001	.10/.31/.58
4	171	192,837.12	193,982.509	193,439.119	0.805	< 0.001	< 0.001	.12/.15/.33/.39
5	214	192,196.131	193,629.541	192,949.509	0.823	< 0.001	< 0.001	.11/.10/.08/.34/.37
6	257	191,593.208	193,314.64	192,497.966	0.776	0.378	< 0.001	.08/.07/.27/.33/.10/.15
7	300	191,223.427	193,232.881	192,279.565	0.735	0.7812	< 0.001	.08/.10/.20/.19/.22/.16/.07

*BIC* Bayesian Information Criterion, *SSA-BIC* Sample-Size Adjusted BIC, *AIC* Akaike Information Criterion, *LMRA-A* Lo-Mendell-Rubin adjusted likelihood ratio test, *BLRT* bootstrap likelihood ratio test

In addition, an LCA for the 13- to 15-year follow-up period was applied to further verify the stability and scientific validity of this classification, as well observe the changes over time in the proportion of different categories of functional ability. As shown in Table 2, the 3-year LCA results showed that entropy is greater than 0.8, indicating that the accuracy of this classification is more than 90%. The values of AIC and BIC are small, indicating that the model fits well. Moreover, the BLRT test was passed in all three years. This illustrates that the number of categories set 5 was appropriate. Furthermore, it was found that the fifth category of older adults with functional limitation had the largest proportion of the population at each time point, although always in a dynamic process of change. The reason behind might be this group can be supplemented by transfers from other functional categories. Thus, the class 5 was considered to be an intermediate status between disability and health, which defined as “sub-disorder status”, with a very large scope for modulation and intervention, and provides important information and is of great relevance for delaying the progression of disability and preventing its occurrence.

**Table 2:** Latent class models with fitted metrics and category probabilities (2011–2015)

Model	Model	AIC	BIC	SSA-BIC	Entropy	LMR-A *p*-value	BLRT *p*-value	Classification probability
2011	5	192,196.131	193,629.541	192,949.509	0.823	< 0.001	< 0.001	.114/.103/.077/.340/.366
2013	5	141,486.905	142,433.500	141,975.915	0.889	< 0.001	< 0.001	.111/.222/.092/.112/.463
2015	5	129,808.661	130,778.856	130,305.386	0.812	0.382	< 0.001	.098/.145/.293/.111/.354

*BIC* Bayesian Information Criterion, *SSA-BIC* Sample-Size Adjusted BIC, *AIC* Akaike Information Criterion, *LMRA-A* Lo-Mendell-Rubin adjusted likelihood ratio test, *BLRT* bootstrap likelihood ratio test

**Fig. 3:**
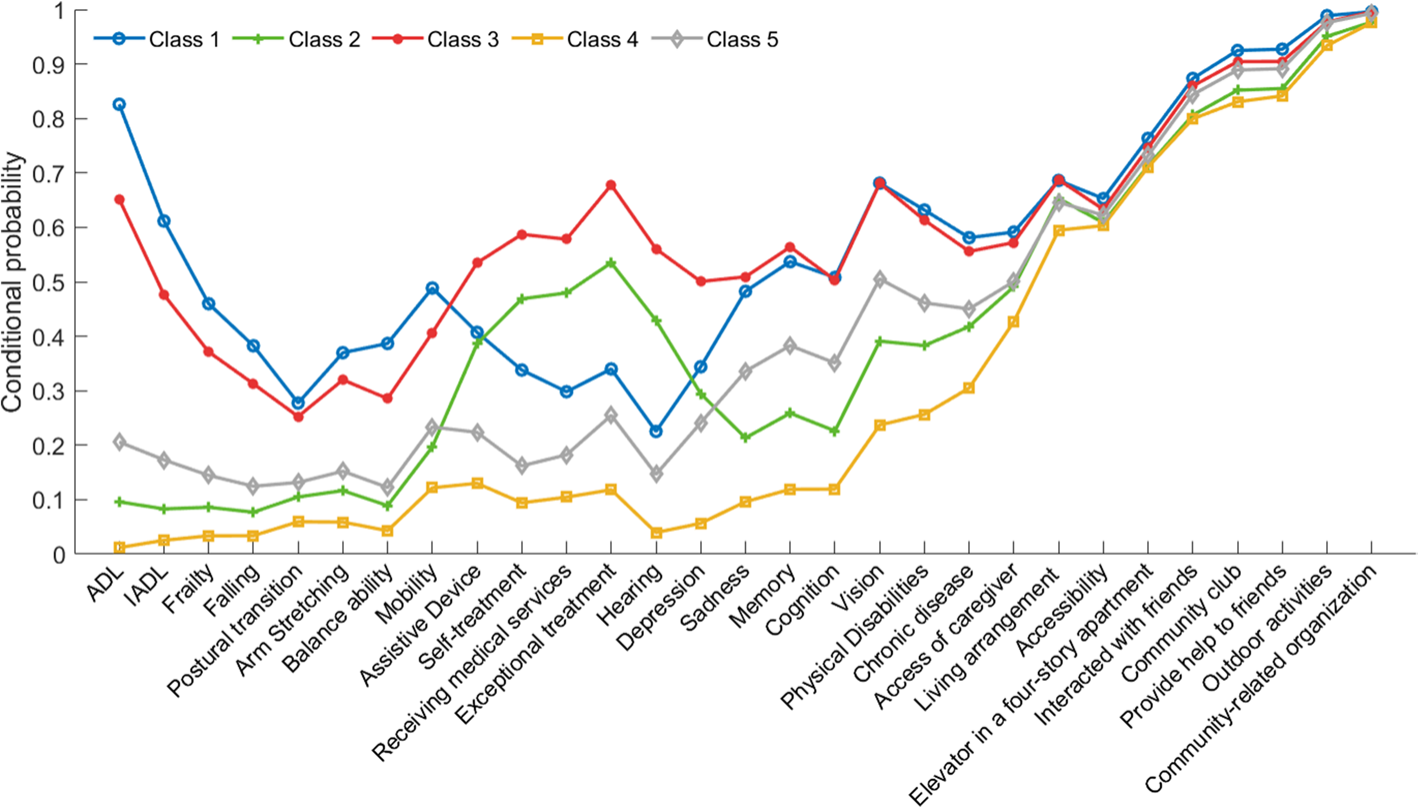
Multidimensional functional indexes of intrinsic capacity, environment and social interaction by class. Note. class 1: viability disorder; class 2: acute disease; class 3: somatic functional disorder; class 4: health; class 5: sub-disorder status

A graphical depiction of conditional probability distribution of multidimensional functional indicators in each category was provided in Fig. 3, and stratified by gender, age and region (Supplementary Figs. S1, S2, S3, S4, S5, S6 and Tables S4, S5, S6). Based on comprehensive analysis of above results, we found that class 1 had a higher dysfunction rate in all items, except self-treatment, receiving medical services, exceptional treatment and hearing condition, as compared to class 2, "health", and "sub-disorder status". Moreover, an LCA for the 13- to 15-year follow-up period showed that both the proportion of older adults with the most severe functional decline in the first category and the best functional conditions in the fourth category decreased over time. The former possibly is due to an increase in mortality, while the latter might result from a transfer to other functional categories. Thus class 1 was named as "viability disorders". Likewise, class 3 had a poor function in all aspects and the severity, which is similar to that of class 1, especially in the medical treatment, the use of auxiliary devices, self-care, motor ability, medical condition, sources of care, home settings, social interactions, thus defined as "somatic functional disorders". Moreover, as these two categories are both in severe impairments in intrinsic capacity (e.g. ADL and IADL impairments), they are close to disability states. Class 2 had higher rates use of auxiliary devices, high probability of seeking medical treatment and special treatment conditions, while other aspects of function are in a good state, which is defined as "acute disease onset". However, the classes of "acute disease onset" and "somatic functional disorder" suggested that functional impairment in self-treatment, receiving medical services, exceptional treatment and hearing condition were both worse than the rest of three categories. The reason behind it might be the individuals in the "acute disease onset" more likely to get an acute injury to seek medical care and lose normal cognitive mental status in a sudden, and close to "viability disorder" for a short time period.

#### Characteristics of five categories

The description of sociodemographic characteristics and health behavior of 5 categories were shown in Table 3 and Fig. S7 in supplement, where “acute disease” class age 67.04 ± 5.98 years, BMI 22.91 ± 3.71 kg/m2, Systolic pressure 132.15 ± 24.93 mm/Hg, Diastolic pressure 74.01 ± 12.33 mm/Hg, hours of informal care 2.15 ± 19.17 h, hours of formal care 6.95 ± 48.15 h, Sleeping duration 6.07 ± 3.47 h), "somatic functional disorders" class (age 69.36 ± 7.25 years, BMI 22.74 ± 4.41 kg/m^2^, Systolic pressure 136.49 ± 23.85 mm/Hg, Diastolic pressure 75.39 ± 12.77 mm/Hg, hours of informal care 16.09 ± 52.7 h, hours of formal care 47.80 ± 95.17 h, Sleeping duration 5.33 ± 2.39 h), "viability disorders" (age 71.54 ± 7.63 years, BMI 22.60 ± 4.50 kg/m2, Systolic pressure 141.56.09 ± 30.50 mm/Hg, Diastolic pressure 76.73 ± 12.70 mm/Hg, hours of informal care 33.73 ± 92.07 h, hours of formal care 83.55 ± 143.61 h, Sleeping duration 5.60 ± 2.41 h), "sub-disorder status" (age 68.25 ± 6.64 years, BMI 22.84 ± 4.23 kg/m2, Systolic pressure 136.30 ± 25.04 mm/Hg, Diastolic pressure 75.00 ± 12.02 mm/Hg, hours of informal care 4.79 ± 29.99 h, hours of formal care 14.85 ± 59.44 h, Sleeping duration 6.09 ± 2.07 h), "health" (age 66.76 ± 5.88 years, BMI 23.04 ± 3.89 kg/m2, Systolic pressure 134.98 ± 24.78 mm/Hg, Diastolic pressure 75.25 ± 11.20 mm/Hg, hours of informal care 0.49 ± 8.17 h, hours of formal care 1.33 ± 14.18 h, Sleeping duration 6.63 ± 1.64 h).

**Table 3:** Participants characteristics among 5 categories

	Class1	Class2	Class3	Class4	Class5
N	685(11.4)	618(10.3)	463(7.7)	2036(34.0)	2190(36.6)
Sex					
Male	254(37.0)	318(51.5)	174(37.6)	1257(61.8)	991(45.2)
Female	431(63.0)	300(48.5)	289(62.4)	779(38.2)	1199(54.8)
Age	71.54 ± 7.63	67.04 ± 5.98	69.36 ± 7.25	66.76 ± 5.88	68.25 ± 6.64
Marital status, %					
Married	463(67.6)	506(81.9)	345(74.5)	1712(84.1)	1679(35.7)
Divorced	217(31.7)	111(18.0)	116(25.1)	305(15.0)	489(22.3)
unmarried	5(0.7)	1(0.2)	2(0.4)	19(38.8)	22(1.0)
Educational level, %					
illiteracy	387(56.5)	185(29.9)	248(53.6)	469(23.0)	923(42.1)
Primary school	239(34.9)	302(48.9)	188(40.6)	992(48.7)	1000(45.7)
High school	54(7.9)	117(18.9)	26(5.6)	507(24.9)	246(11.2)
Bachelor and above	5(0.7)	14(2.3)	1(0.2)	68(3.4)	21(1.0)
Place of residence					
Rural	583(85.1)	481(77.8)	427(92.2)	1443(70.9)	1832(83.7)
Urban	102(14.9)	137(10.3)	36(7.8)	593(29.1)	358(16.3)
Numbers of children, %					
0	177(25.8)	158(25.6)	122(26.3)	572(28.1)	633(28.9)
1 ~ 3	168(24.5)	203(32.8)	106(22.9)	753(37.0)	639(29.2)
4 ~ 5	220(32.1)	192(31.3)	156(33.7)	515(25.3)	629(28.7)
> 5	120(17.5)	65(10.5)	79(17.1)	196(9.6)	289(13.2)
BMI	22.60 $\pm$ 4.50	22.91 $\pm$ 3.71	22.74 ± 4.41	23.04 ± 3.89	22.84 ± 4.23
Systolic pressure	141.56.09 ± 30.50	132.15 ± 24.93	136.49 ± 23.85	134.98 ± 24.78	136.30 ± 25.04
Diastolic pressure	76.73 ± 12.70	74.01 ± 12.33	75.39 ± 12.77	75.25 ± 11.20	75.00 ± 12.02
Sleeping duration	5.60 ± 2.41	6.07 ± 3.47	5.33 ± 2.39	6.63 ± 1.64	6.09 ± 2.07
Hours of informal care	33.73 ± 92.07	2.15 ± 19.17	16.09 ± 52.7	0.49 ± 8.17	4.79 ± 29.99
Hours of formal care	83.55 ± 143.61	6.95 ± 48.15	47.80 ± 95.17	1.33 ± 14.18	14.85 ± 59.44
Satisfaction					
Extremely	8(1.2)	8(1.3)	3(0.6)	53(2.6)	27(0.5)
Very much	97(14.2)	126(20.4)	61(13.2)	497(24.4)	427(19.5)
Relative	430(62.8)	419(67.8)	296(63.9)	1346(66.1)	1449(66.2)
A little	114(16.6)	58(9.4)	74(16.0)	130(6.4)	237(10.8)
Not at all	36(5.3)	7(1.1)	29(6.3)	10(0.5)	50(2.3)
Self-rated health					
Very good	4(0.6)	10(1.6)	2(0.4)	145(7.1)	45(2.1)
Good	25(3.6)	52(8.4)	13(2.8)	455(22.3)	205(9.4)
Fair	174(25.4)	264(42.7)	110(23.8)	1072(52.7)	1055(48.2)
Bad	317(46.3)	235(38.0)	224(48.4)	338(16.6)	717(32.7)
Very bad	165(24.1)	57(9.3)	114(24.6)	26(1.3)	168(7.7)

class 1: viability disorders, class 2: acute disease, class 3: somatic functional disorders, class 4: health, class 5: sub-disorder status

#### Differences for trend across five categories over time

ANOVA and Chi-square analyses were performed to assess differences in sociodemographic characteristics, biological factors and health behavior across the classes identified in the LCA. The results of Table S7 in the supplement indicated that the five classes differed by age, gender, educational level, marital status, blood pressure, sleeping duration, life satisfaction, and self-rated health. In addition, Fig. S8 in supplement presented that the distribution of those major characteristics of the five categories changed synchronously with time, indicating that the classification results were relatively stable. The "somatic functional disorders" and "viability disorders" classes were in a higher proportion of females and older ages, higher blood pressure, shorter sleep duration, longer formal care duration, poorer life satisfaction, and higher rates of poor self-reported health status than other groups. In contrast, the proportion of men in the "health" class with the normal function was the highest. Notably, the proportion of illiteracy in the "viability disorders" was the highest, and the proportion of older adults with more than 5 children was the highest. It also can be seen from the supplementary Fig. S9 that the proportion of "somatic functional disorders", "viability disorders" and "sub-disorder status" among elderly female aged 70 and above who lived in the rural areas was relatively higher than males under 70 and living in cities and towns.

#### Development and validation of functional classification

**Fig. 4:**
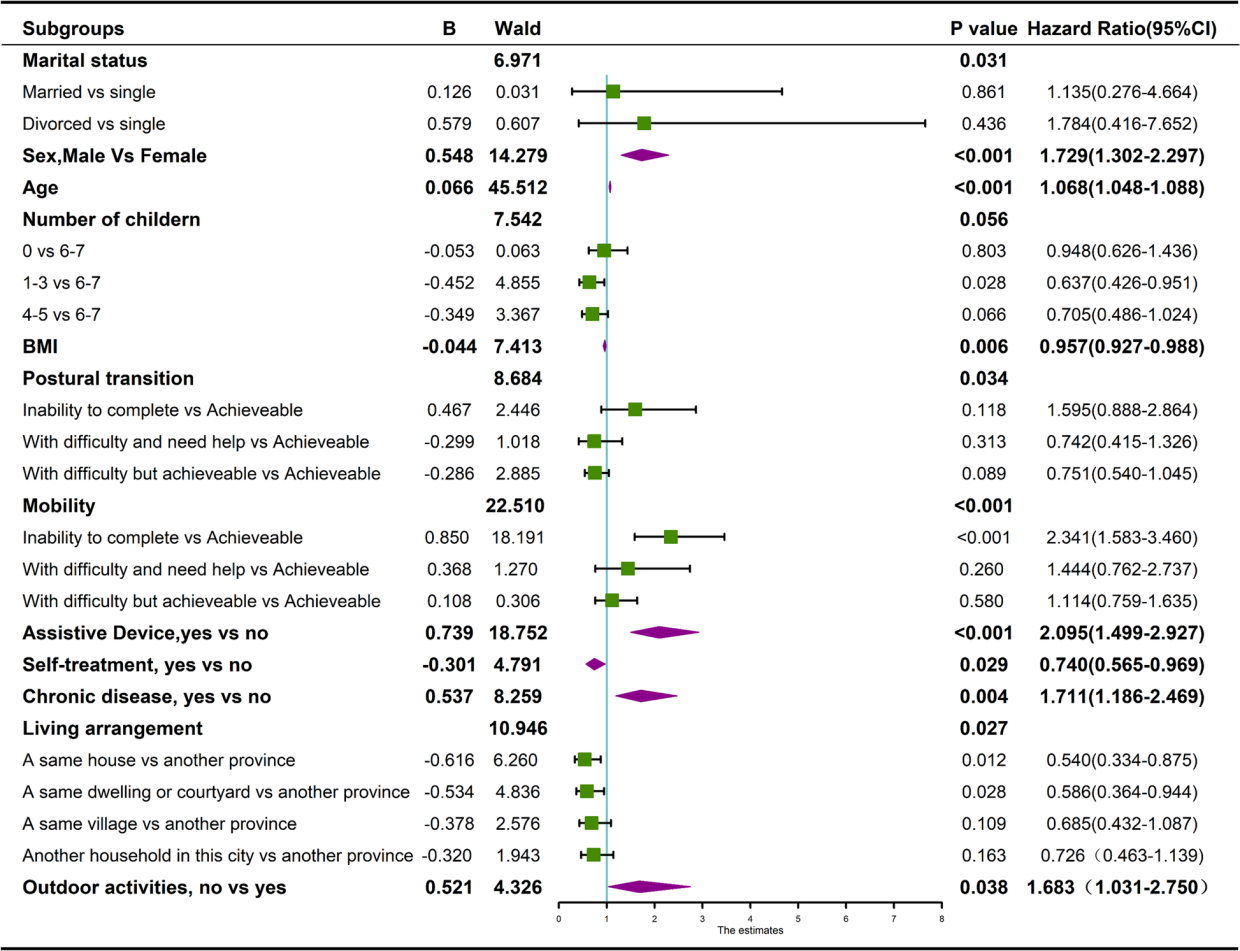
Forest plots of overall survival according to stratified variables and sociodemographic characteristics during follow-up. Note. B = Unstandardized β coefficients were calculated from the Multiple Cox regression analysis

The Multiple Cox regression analysis showed that after adjusting for the baseline characteristics, the effects of stratified variables on survival remained consistent with the primary analysis. The results of the univariate logistic analysis are presented in supplementary Table S8. On this model, with reports as hazard ratio (95% CI); male (HR 1.73, 95% CI 1.30–2.30; *p* < 0.001 vs female); with 1–3 children (0.64, 0.43–0.93; *p* = 0.028 vs with 6–7 children); inability to mobility(2.34, 1.58–3.46; *p* < 0.001 vs achievable mobility); use of assistive device(2.10, 1.50–2.93; *p* < 0.001 vs without use of assistive device); self-treatment (0.74, 0.56–0.97; *p* = 0.029 vs good cognition), chronic disease(1.71, 1.19–2.47;*p* = 0.004 vs without chronic disease), living in a same house with children (0.54, 0.33–0.88; *p* = 0.012 vs living in another province), living in a same dwelling or courtyard (0.57, 0.36–0.94; *p* = 0.028 vs living in another province), were independently associated with mortality (Fig. 4).

#### Survival analysis

**Fig. 5:**
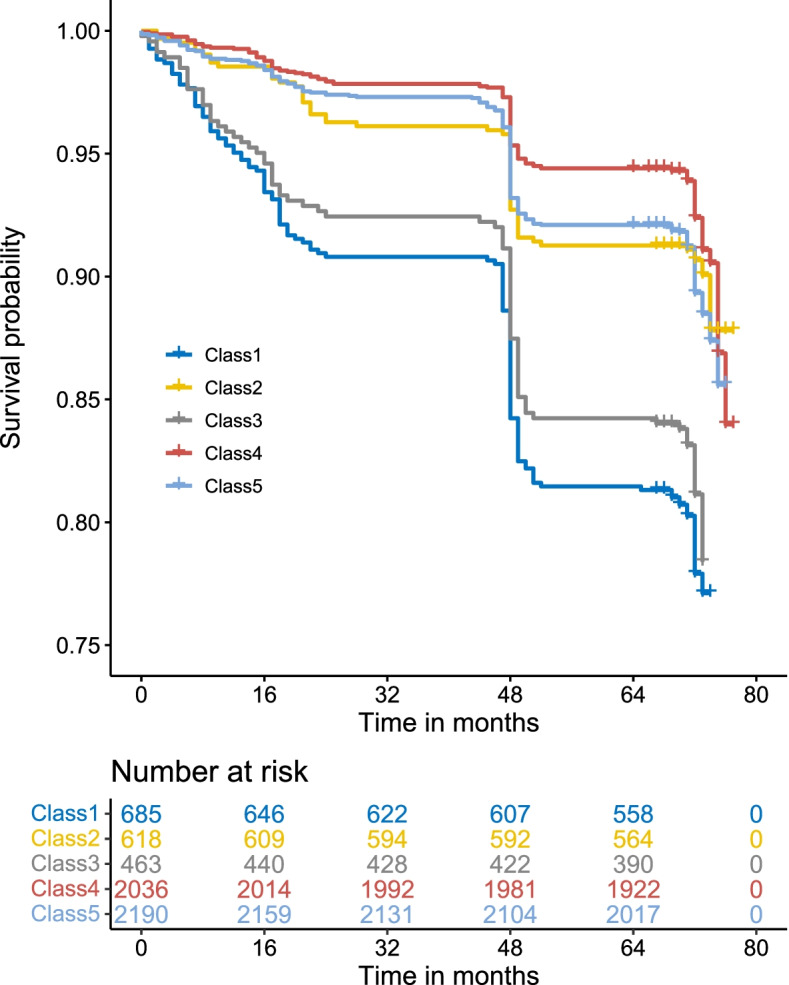
Survival progression according to the new functional classification during follow-up (Note. Cox proportional hazard model adjusted for baseline characteristics; class 1: viability disorders; class 2: acute disease; class 3: somatic functional disorders; class 4: health; class 5: sub-disorder status)

All variables used in this analysis were based on the 29 variables which used to construct a multi-dimensional functional indexes and the sociodemographic Characteristics. The results presented that the risk of mortality gradually increased toward the less functionally independent end of the classification. This indicates that the elucidation of a new functional classification composed of intrinsic capacity, environments, and social interaction indicators, is effective in stratifying the risk for mortality in older adults. The presence of impairment was associated with mortality (*p* < 0.001), in model adjusted for age, sex, marital status, place of residence, household per capita income, BMI, educational level and numbers of children (Fig. 5). To account for the differences in 7-year survival rates between the categories of the functional classification, survival curves were fitted. The overall mortality in 5992 older adults was 3.8%. Thirty-three of 685 older adults in the “viability disorder” class, 24 of 618 in “acute disease” class, 63 of 463 in “somatic functional disorder”, 44 of 2036 in “health”, 59 of 2190 in “sub-disorder status”, died within 7 years. The comparison of the survival differences between different categories suggested that the “health” and “sub-disorder status” survival was prolonged compared with “viability disorder” (*P* < 0.001), while there was no significant difference of “acute disease” and “somatic functional disorder” survival compared with “viability disorder” (Table S10 in supplement).

#### Analysis of ADLs impairments

The risk of 7-year ADLs impairments gradually increased toward the less functionally independent end of the classification, which is close to mortality patterns. The presence of functional decline among categories was associated with the presence of ADLs impairments, in models adjusted for baseline characteristics (Fig. S10 in supplement). To illustrate the differences in 7-year ADLs impairment rates among the functional categories, hazard curves were fitted. The overall ADLs impairment rate for 3971 older adults was 49.2%. Seventy of 82 older adults in “viability disorder”, 242 of 469 in “acute disease”, 93 of 123 in “somatic functional disorder”, 603 of 1695 in “health”, 896 of 1501 in “sub-disorder status”, got ADLs impairments within 7 years. The comparison of the differences in ADLs impairments of different categories suggested that the survival period of “sub-disorder status”, “health” and “acute disease” was prolonged compared with that of “viability disorder” (*P* < 0.001), but there was no significant difference of “somatic functional disorder” in ADLs impairments compared with “viability disorder” (Tables S9 and S10 in supplement).

#### Competitive risk modeling

The results of the competitive risk regression analysis showed that Log pseudolikelihood = -3012.434, Wald = 169.65, *P* < 0.001. There is a competing risk of accidental death with the ADLs outcome. Taking the category variables as an example, after controlling for sociodemographic variables such as age, gender, and marital status, the risk of ADLs impairments in class 2, class 4 and class 5 had 0.536, 0.268, and 0.514 times the risk of ADLs impairments than class 1, respectively, with a statistically significant difference (*P* < 0.05). This showed a consistency with the Cox risk regression, and it is a sensitivity analysis to further support our conclusions (Table S11 in supplement).

## Discussion

Taken together, the results of this study showed that new classification of older adults with functional limitations was superior to the classic functional clusters because it initially identifies high-risk functional decline with limited intrinsic capacity, environments and social interaction, and provided information about underlying disability to guiding choice of intervention. Unlike previous attempts to dissect the functional heterogeneity of older adults [43], we applied comprehensive variables of key aspects of functional ability monitored in the older population. Therefore, this clustering can be easily applied to both existing functional independence cohorts (eg, from sports intervention), especially for older people in the home-cased community. An assessment tool for assigning the older people to specific clusters was being developed, provided that the appropriate variables have been measured.

A new functional classification effectively stratified the risk for mortality in older adults. Our classification composed of 8 dimensions and 29 indexes. As 5 exclusive categories representing the functional continuum, it can effectively stratify the risk for mortality in older adults, that is independent of age, sex, marital status, place of residence, household per capita income, BMI, educational level and numbers of children. These categories coming from a population-based study are not only an epidemiologic structure but may also help clinicians to implement diagnostic and therapeutic procedures, advanced care planning, and assign resources to older adult in the entire functional continuum, starting from normal function (category 4) to functional decline (categories 2 and 5), and then to disability (categories 1 and 3). Since disability is a common end-result of acute and chronic conditions in the older population [44, 45], the new classification could help solve the always difficult decision process around when to screen and take the subsequent interventions for osteoarthritis [46], depression [47], adverse events [48] and a major predictor to the years to survive, which now is age-determined. Other procedures that could benefit from the use of the new classification in the treatment decision-making process might be heart failure, chronic kidney disease, among others. Our work could be considered as a proof of concept, since the functional categories work across physical condition, care sources, different settings and social support, which was less taken into account previously.

The results presented that functional impairments in social support, home settings, sources of care, medical condition, movement, ability of processing diseases, cognition and communication, and self-care are health conditions independently associated with mortality. It is well known that the limitations of ADLs and IADLs are not the only two independent factors that identify the risk of other health-related adverse consequences for older adults, as well as institutionalization [49], hospitalization [49],quality of life [50], and health care costs [51]. Furthermore, in agreement with WHO healthy ageing framework we have described in previous work that the multidimensional functional index is the better predictor of adverse events in older adults, more predictive than ADLs or IADLs, and should be considered the cornerstone of geriatric medicine.

Other procedures could benefit from the use of the new classification in the decision-making of relevant policy implications, since health care and social service decisions may be adopted on the basis of validated stratification [52]. Our work could be considered as a proof of concept; In this category, the “health” category in the new classification is similar to the WHO’s definition of health as “a state of complete physical, mental, and social well-being and not merely the absence of disease or infirmity” [53]; those in “sub-disorder status” is considered to be an intermediate status between disability and “health”, with a very large scope for modulation and intervention, and provides important information and is of great relevance for delaying the progression of disability and preventing its occurrence. However, “acute disease” class has a similar mortality risk as those that are “sub-disorder status”, and should be managed by primary care to take timely treatment and postoperative rehabilitation training to avoid the processing toward “viability disorder”, “somatic functional disorder” and “sub-disorder status”.

Thereafter, the high mortality and other risks of adverse outcomes of participants who got poor performances temporarily, but got better in other dimensions could make us include this category as a unique one “somatic functional disorder” coexists with the highest impairment on cognitive mental status and communication skills, and ability of processing diseases, but with lower self-care limitation than “viability disorder”. Older adults often relapse after treatment with a high disability rate [54]. The rehabilitation process after treatment should be a highlight to avoid permanently damaged in this group. "viability disorder" class was severely damaged in self-care, and it was unlikely to get better and move to other categories. The “somatic functional disorder” has more chronic diseases, such as arthritis, dementia and diabetes, indicating the need of more treatment [55, 56], while the "viability disorders" had more lethal diseases, including heart diseases and stroke, and demonstrating less period of hospitalization [57], which partly explains the longer but more morbid survival of “somatic functional disorder”. Therefore, the existing long-term care insurance should pay the most attention to those who lived longer, have complex but non-fatal health problems and need long-term intensified treatment, which consumes resources and lays a heavy burden on family and society.

Additionally, a subset of women, who had more children, with less education, higher blood pressure, longer sleep time, longer care time, and poorer life satisfaction are more likely to posted in “viability disorder” and “somatic functional disorder”. Special attention needs to be paid to these two categories of female elderly to improve their health literacy. For example, it is recommended that the government specifically set up community education activities, vital signs health monitoring facilities and public service activities for older adults with high risk of severe disability categories in order to shift to a better functional status and delay the progression of disability.

Nevertheless, there existed three limitations to this study that need to be addressed: the first important one is that it needs external validation in other countries and settings, although categories were created based on statistical analysis, latent class analysis and geriatric medicine knowledge and were thought to be a well-based proof of concept. The second is the hierarchical structure of these 5 categories of functional ability is itself controversial. However, we have adopted survival analysis and logistics regression analysis to explore the heterogeneity among the 5 categories to provide objective evidence, which may be more convincing. Furthermore, taking competing risks into account in the survival analysis of ADLs impairment, we applied compete risk model as a sensitivity analysis, to further support our conclusion. The third limitation is the exact overlap of weaker association signals and transition probability between categories needs to further explored. Nonetheless, an LCA for the 11- to 15-year follow-up period was added to observe the changes over time in the proportion of different functional categories. We consider to use empirical variables in larger cohorts to find out the possible relationships between categories in the future.

## Conclusion

The combined multiple variables central to the development of functional independence is superior to measurement of only one or two aspects, ADL or IADL. The results showed that impaired self-care contributes to the highest mortality risk for older adults and should be regarded as the decisive factor in defining disability level. The “sub-disorder status” was considered to be an intermediate status between disability and health, with a very large scope for modulation and intervention, and provides important information and is of great relevance for delaying the progression of disability and preventing its occurrence. “Acute disease” should be given an urgent primary care to prevent the processing towards "viability disorders" or "somatic functional disorders" class. Close attention must be paid to the health and functioning of the "somatic functional disorders", so as to provide a sound and sustainable basis for planning health. Since the concept of functional decline is closely related to the deprivation of capabilities through restrictions on physical condition, environment and social interaction, based on capacity approach, this study provides a first step representing an important step towards a more precise and useful stratification for older adults in home-based community. Our classification also paves the way for analyzing the care needs and costs of different categories of older people and their future transferred probability among different clusters.

## Supplementary Information

**Additional file 1.**

## Data Availability

CHARLS aims to set up a high quality, nationally representative and publicly available micro-database that provides a wide range of information about the households of the elderly and individual information on the older respondents and their spouses. CHARLS provides broad data that allows for analysis by multiple disciplines. All data stripped of private identifying information will be available for research use at no charge. National baseline data and a baseline national report are public on the CHARLS website and available from http://www.isss.pku.edu.cn/sjsj/charlsxm/index.htm.

## Abbreviations

ADL: Activities of Daily Living
IADL: Instrument Activity of Daily Living
BMI: Body Mass Index
LCA: Latent class analysis
CHARLS: China Health and Retirement Longitudinal Study
AIC: Akaike Information Criterion
BIC: Bayesian information criterion
BLRT: Bootstrap Likelihood Ratio Test
LMR-A: Lo-Mendell-Rubin adjusted
