# Supplementary material for: Novel subgroups of functional ability in older adults and their associations with adverse outcomes
Source: BMC Geriatr. 2022 May 4;22:390. doi: 10.1186/s12877-022-03081-9 (PMC9066860; doi:10.1186/s12877-022-03081-9)
Supplement: Supplementary file 1 — Additional file 1. [file 12877_2022_3081_MOESM1_ESM.docx]

**Supplementary appendix**

**Contents**

**Supplementary Figures**

[Fig. S1. Multidimensional functional ability indexes of intrinsic capacity, environments and social interaction by class (Male) 3](#_Toc100354718)

[Fig. S2. Multidimensional functional ability indexes of intrinsic capacity, environments and social interaction by class (Female) 4](#_Toc100354719)

[Fig. S3. Multidimensional functional ability indexes of intrinsic capacity, environments and social interaction by class (Less than 70) 5](#_Toc100354720)

[Fig. S4. Multidimensional functional ability indexes of intrinsic capacity, environments and social interaction by class (Older than 70) 6](#_Toc100354721)

[Fig. S5. Multidimensional functional ability indexes of intrinsic capacity, environments and social interaction by class (Rural) 7](#_Toc100354722)

[Fig. S6. Multidimensional functional ability indexes of intrinsic capacity, environments and social interaction by class (Urban) 8](#_Toc100354723)

[Fig. S7. The distribution of basic characteristics in terms of sleep duration, age, BMI and blood pressure for different subcategories 10](#_Toc100354724)

[Fig. S8. The distribution of the several major characteristics of the five categories over time (2011 to 2015). 12](#_Toc100354725)

[Fig. S9. The distribution of functional categories in different sex, age and region 12](#_Toc100354726)

[Fig. S10. The progression of ADL impairment over time by categories 13](#_Toc100354727)

**Supplementary Tables**

[Table. S1. The selection and assigned values in 29 indicators 14](#_Toc100354728)

[Table S2. The distribution of functional categories by traditional classification 19](#_Toc100354729)

[Table. S3. Conditional probability distributions of multidimensional functional categories 19](#_Toc100354730)

[Table. S4. Analysis of LCA of sex-stratified functional ability 21](#_Toc100354731)

[Table. S5. Analysis of LCA of age-stratified functional ability 24](#_Toc100354732)

[Table. S6. Analysis of LCA of region-stratified functional ability 26](#_Toc100354733)

[Table. S7. Participants characteristics among 5 categories 29](#_Toc100354734)

[Table. S8. Univariate Logistic Regression Analysis of mortality presence based on baseline Data 31](#_Toc100354735)

[Table S9. Univariate Logistic Regression Analysis of ADLs impairment presence based on baseline Data. 34](#_Toc100354736)

[Table S10. Cox regression analysis comparing mortality and ADLs impairments among categories 37](#_Toc100354737)

[Table S11. Competitive risk modeling of the incidence of ADLs impairments in different categories 38](#_Toc100354738)

**Novel subgroups of functional ability in older adults and their associations with adverse outcomes**


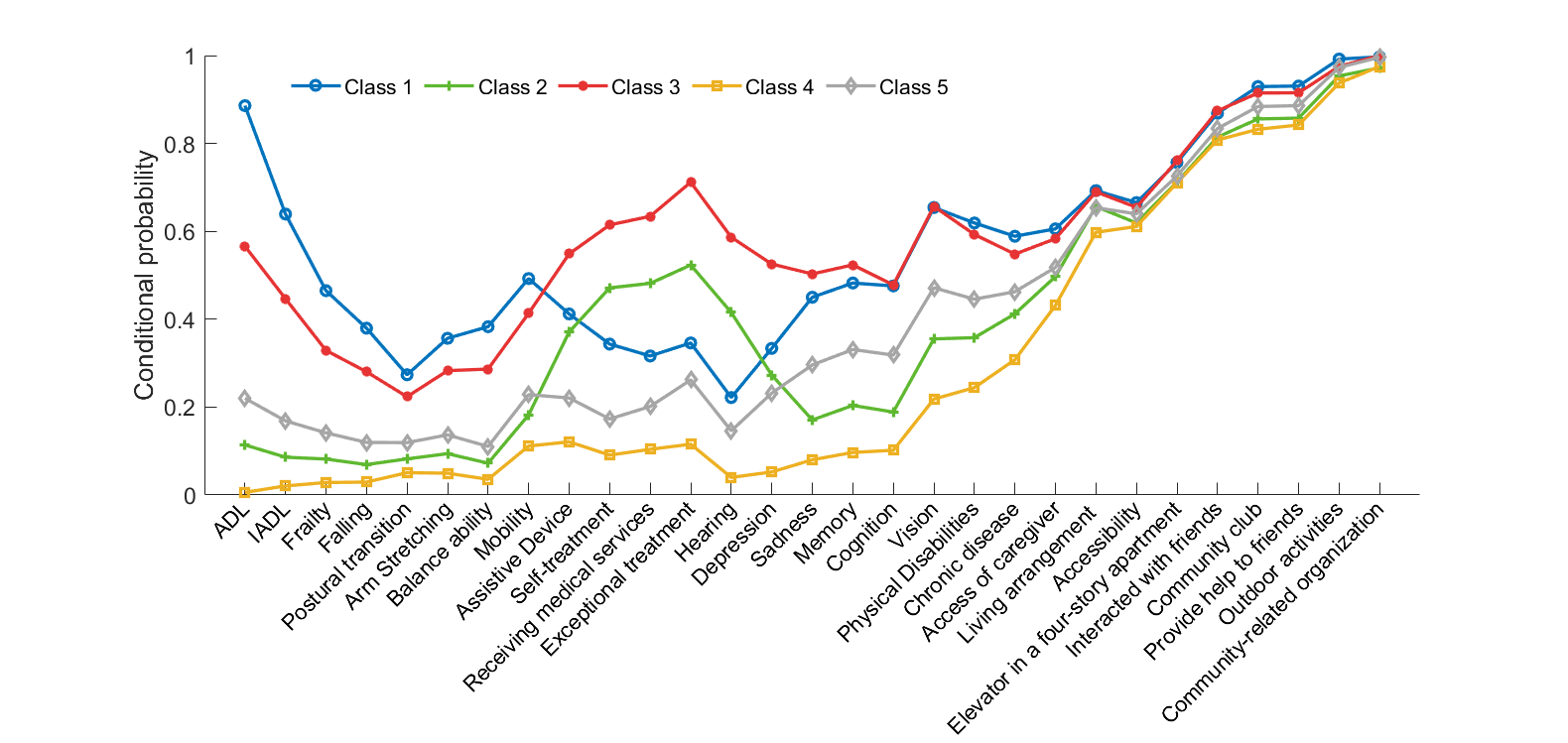


## Fig. S1. Multidimensional functional ability indexes of intrinsic capacity, environments and social interaction by class (Male)

*Note*. class 1: viability disorder; class 2: acute disease; class 3: somatic functional disorder; class 4: health; class 5: sub-disorder status.


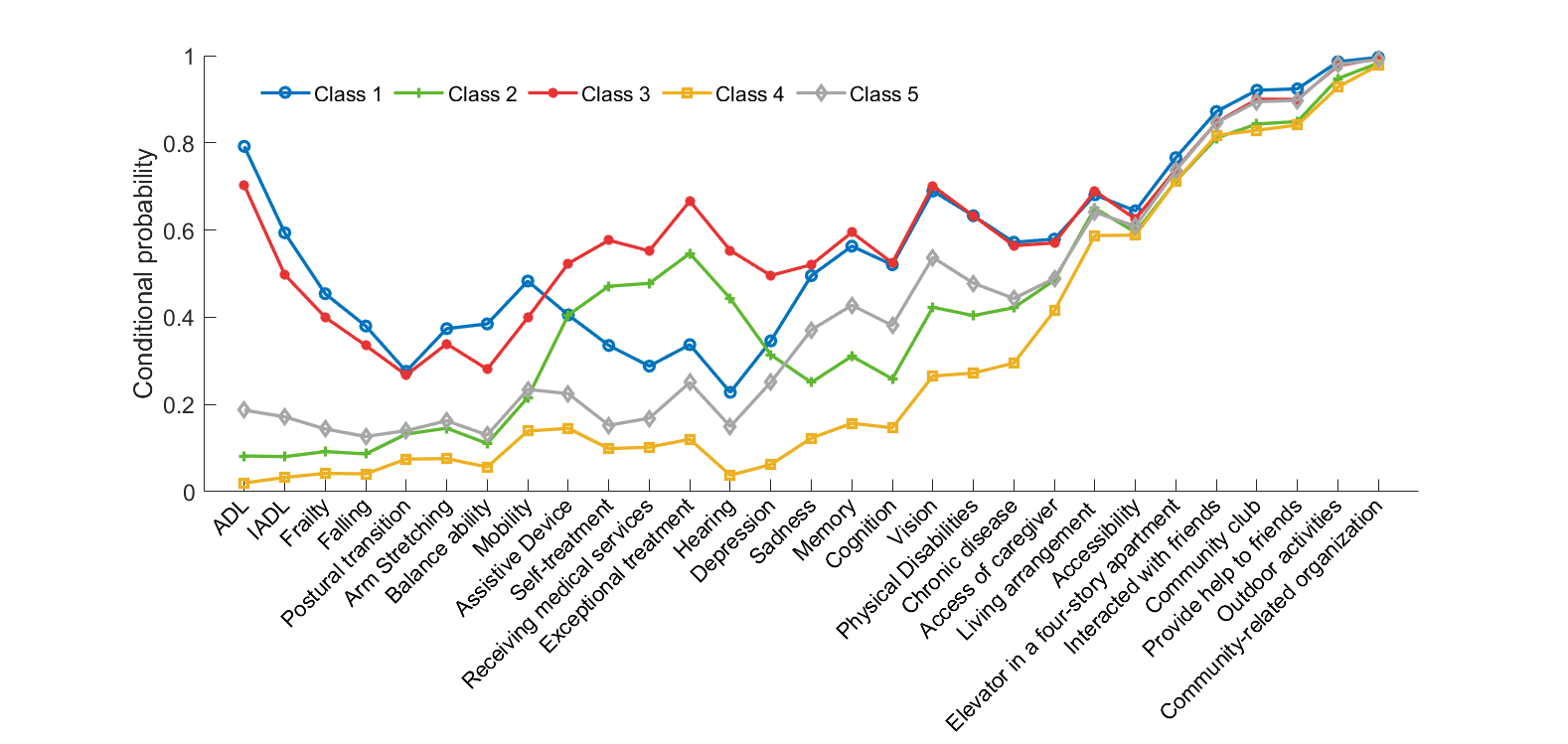


## Fig. S2. Multidimensional functional ability indexes of intrinsic capacity, environments and social interaction by class (Female)

*Note*. class 1: viability disorder; class 2: acute disease; class 3: somatic functional disorder; class 4: health; class 5: sub-disorder status.

**
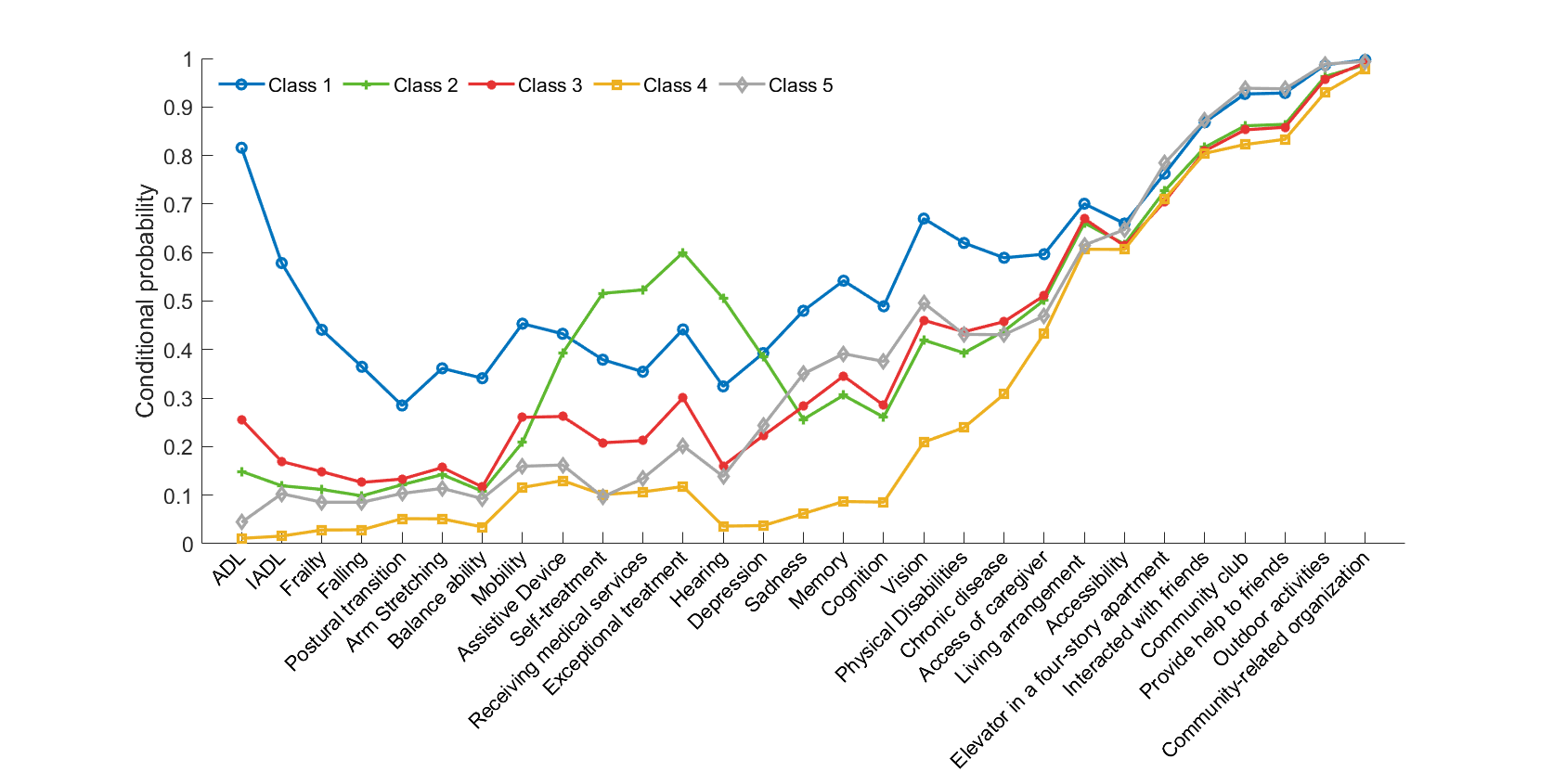
**

## Fig. S3. Multidimensional functional ability indexes of intrinsic capacity, environments and social interaction by class (Less than 70)

*Note*. class 1: viability disorder; class 2: acute disease; class 3: somatic functional disorder; class 4: health; class 5: sub-disorder status.

**
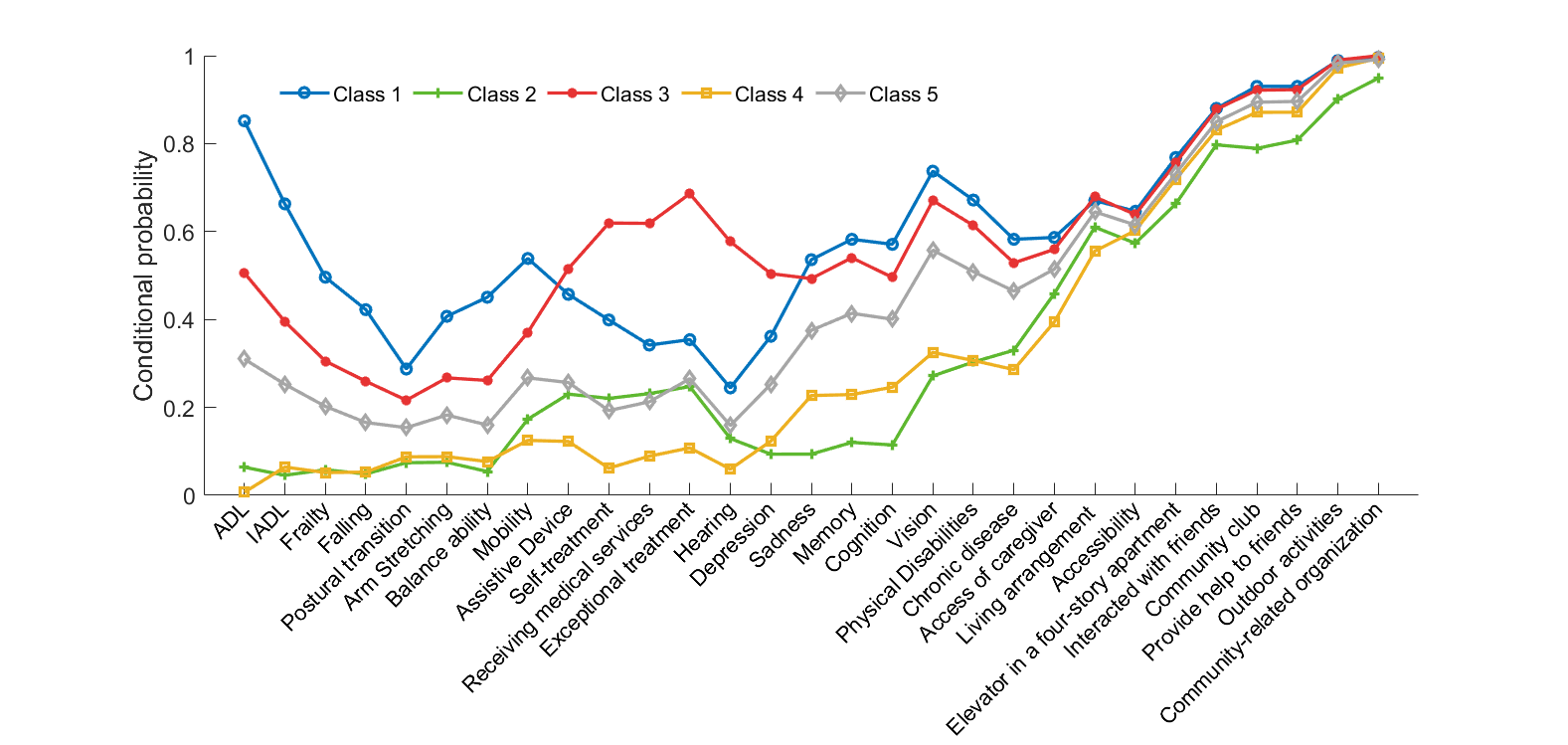
**

## Fig. S4. Multidimensional functional ability indexes of intrinsic capacity, environments and social interaction by class (Older than 70)

*Note*. class 1: viability disorder; class 2: acute disease; class 3: somatic functional disorder; class 4: health; class 5: sub-disorder status.

**
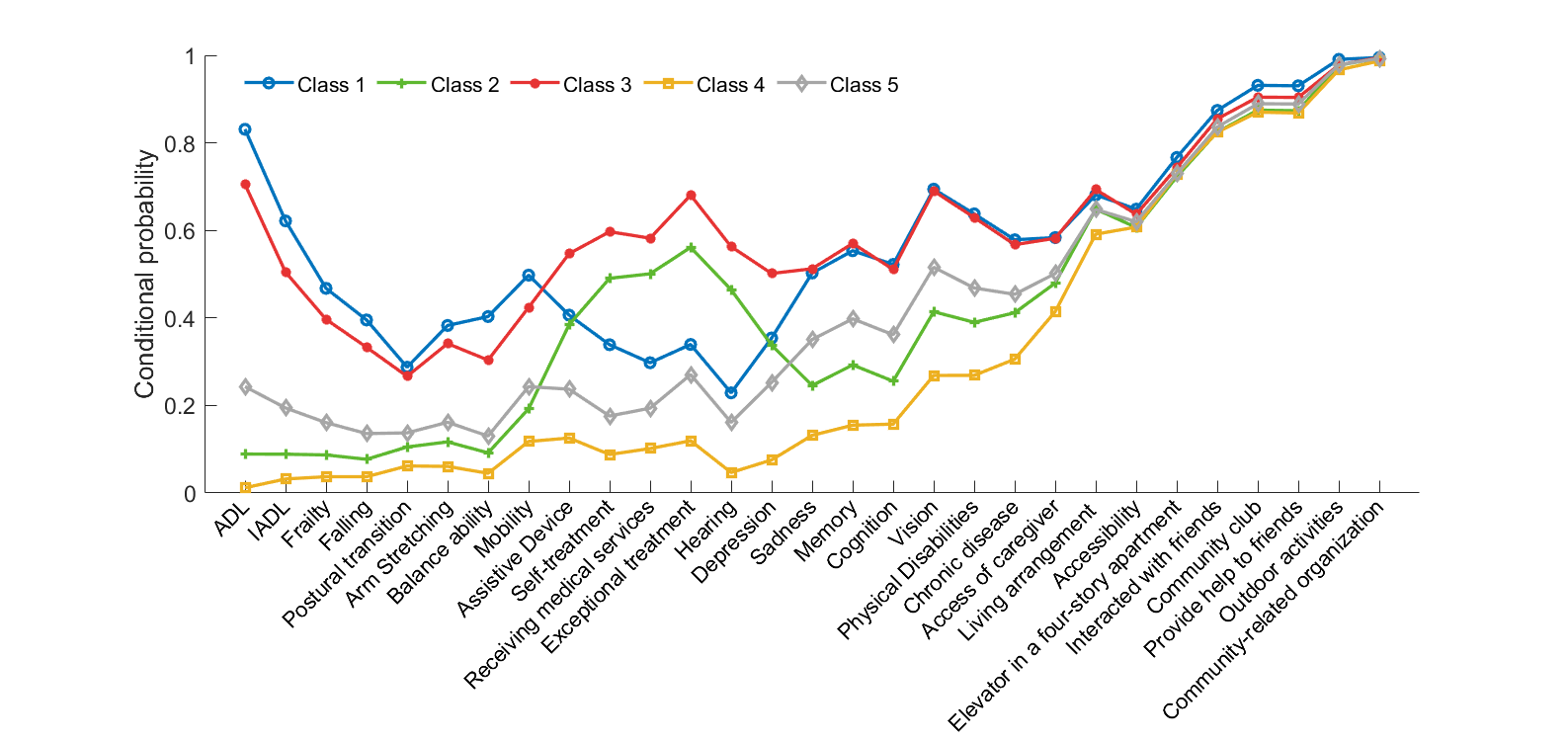
**

## Fig. S5. Multidimensional functional ability indexes of intrinsic capacity, environments and social interaction by class (Rural)

*Note*. class 1: viability disorder; class 2: acute disease; class 3: somatic functional disorder; class 4: health; class 5: sub-disorder status.

**
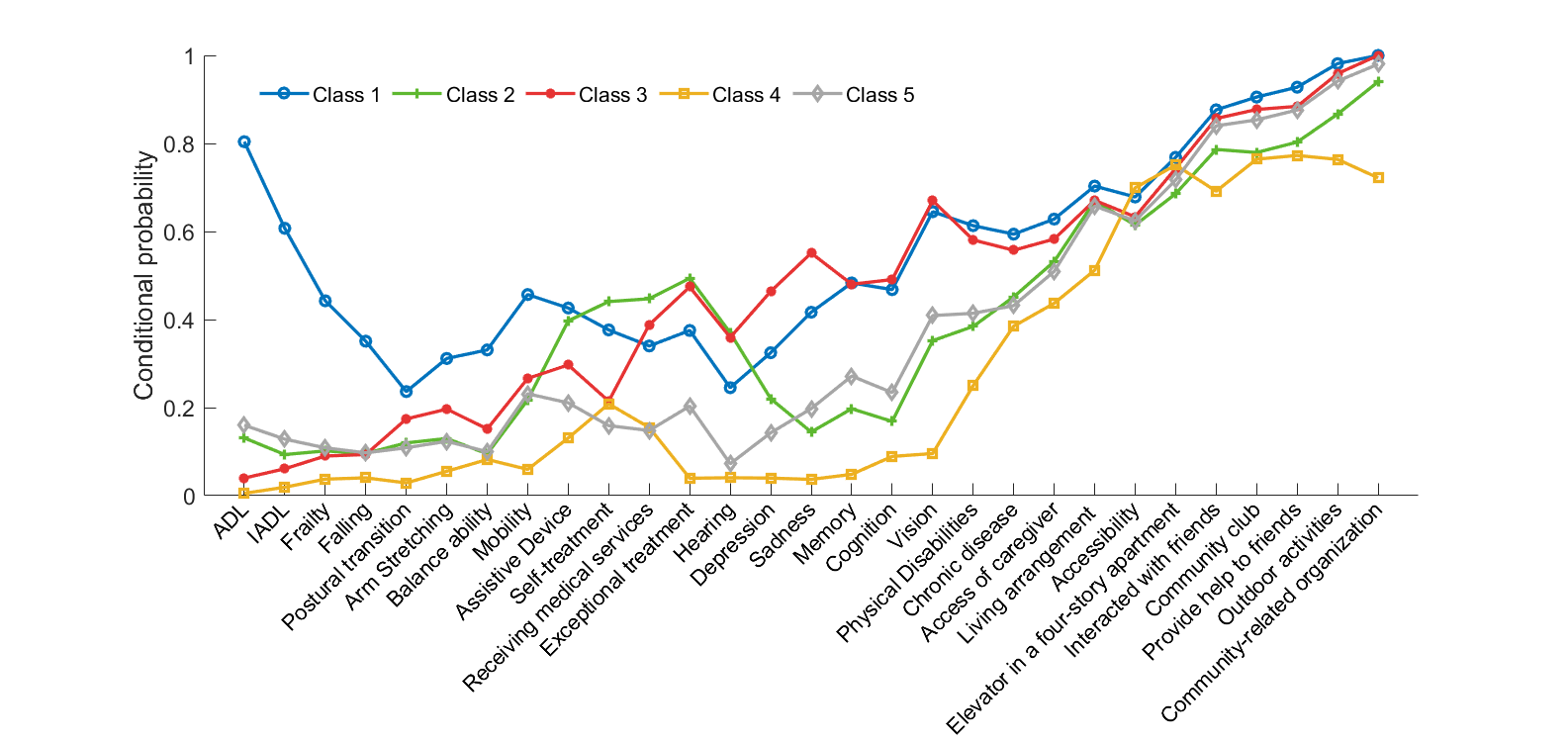
**

## Fig. S6. Multidimensional functional ability indexes of intrinsic capacity, environments and social interaction by class (Urban)

*Note*. class 1: viability disorder; class 2: acute disease; class 3: somatic functional disorder; class 4: health; class 5: sub-disorder status.


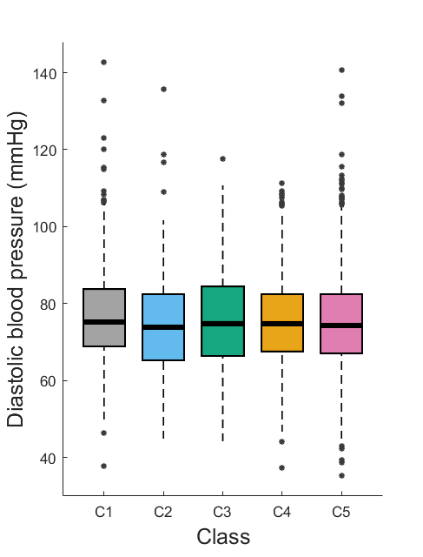

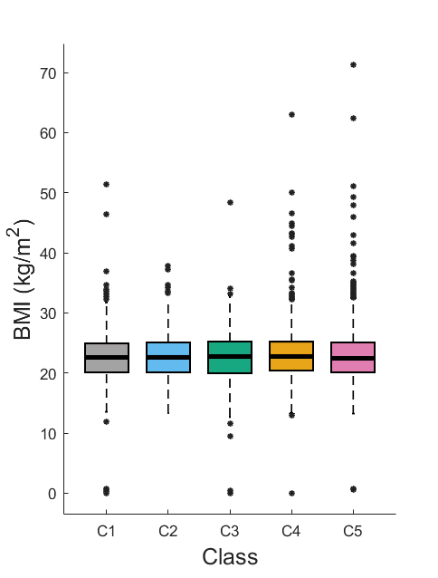

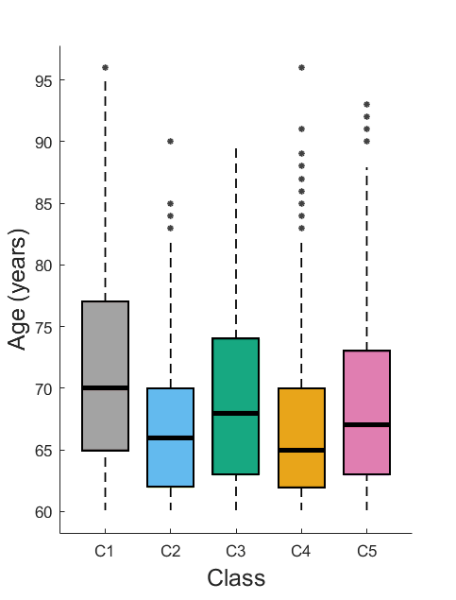


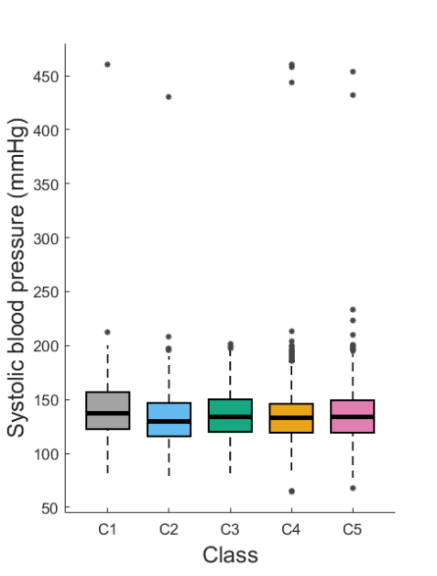


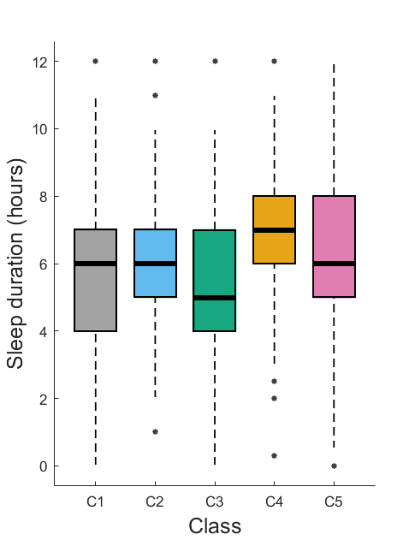


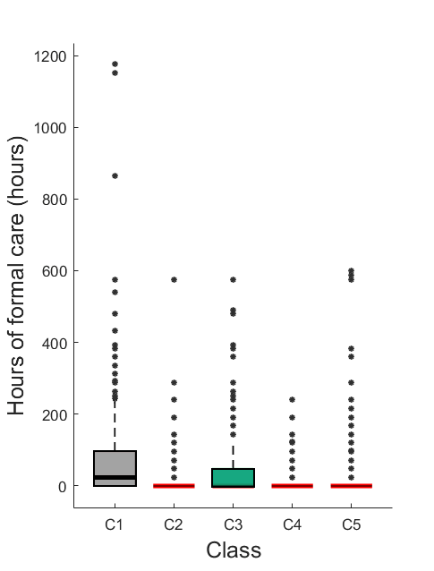

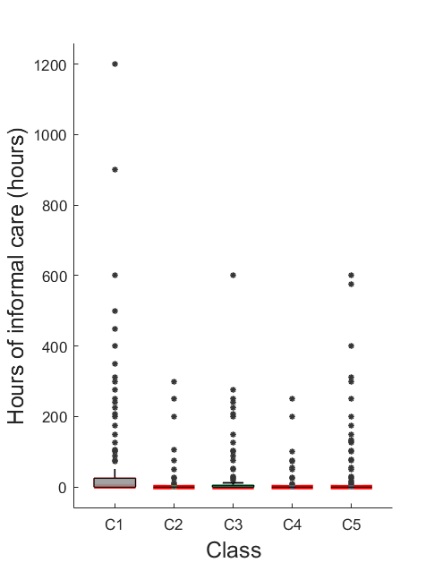


## Fig. S7. The distribution of basic characteristics in terms of sleep duration, age, BMI and blood pressure for different subcategories


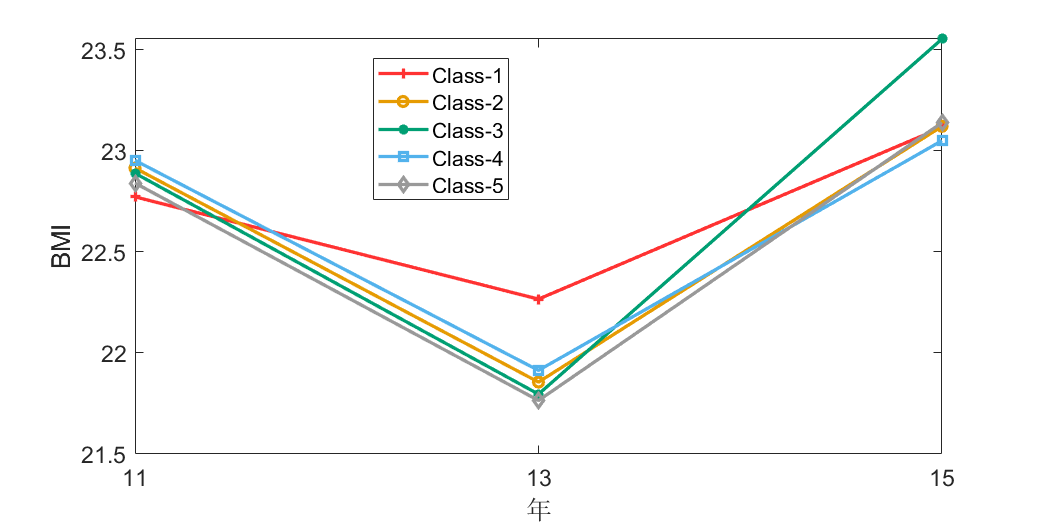


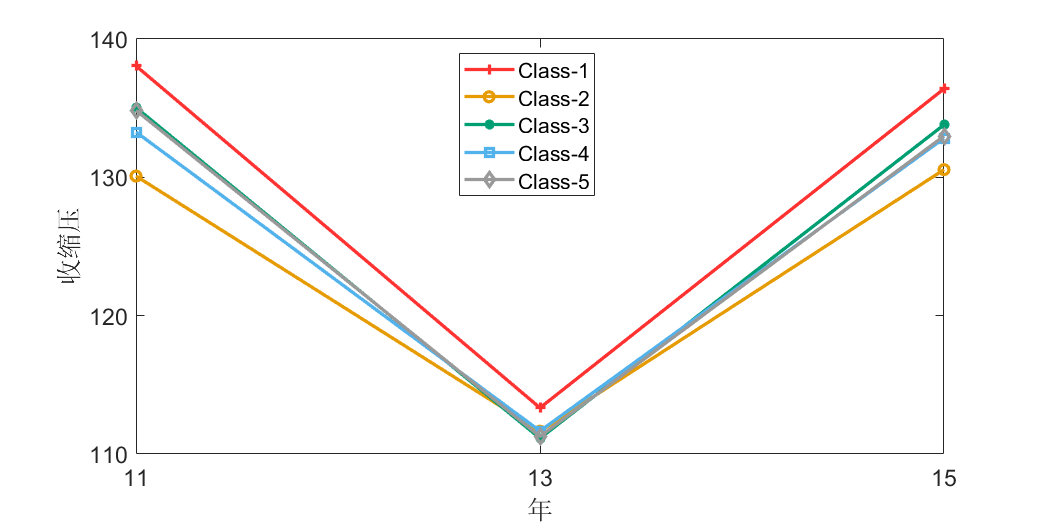


Systolic pressure


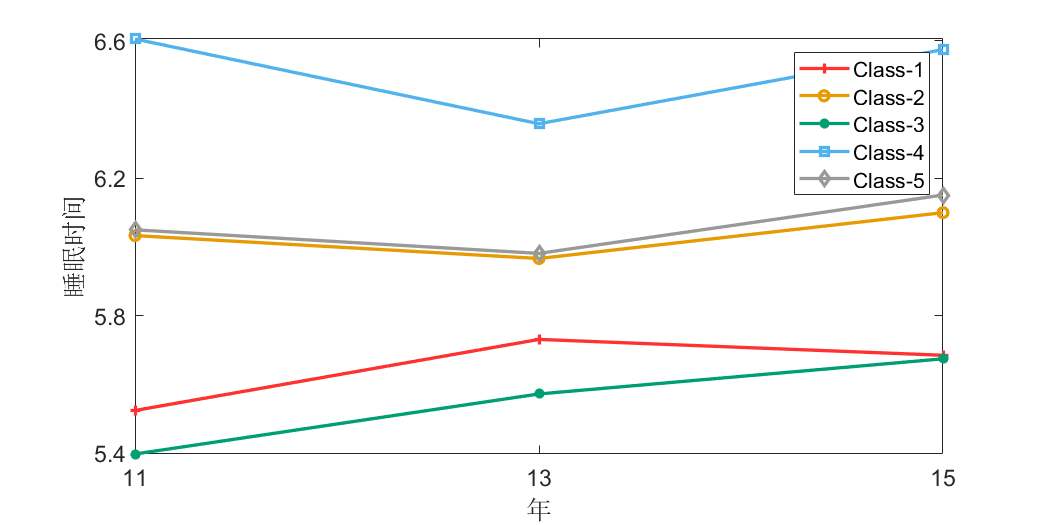


Sleeping duration


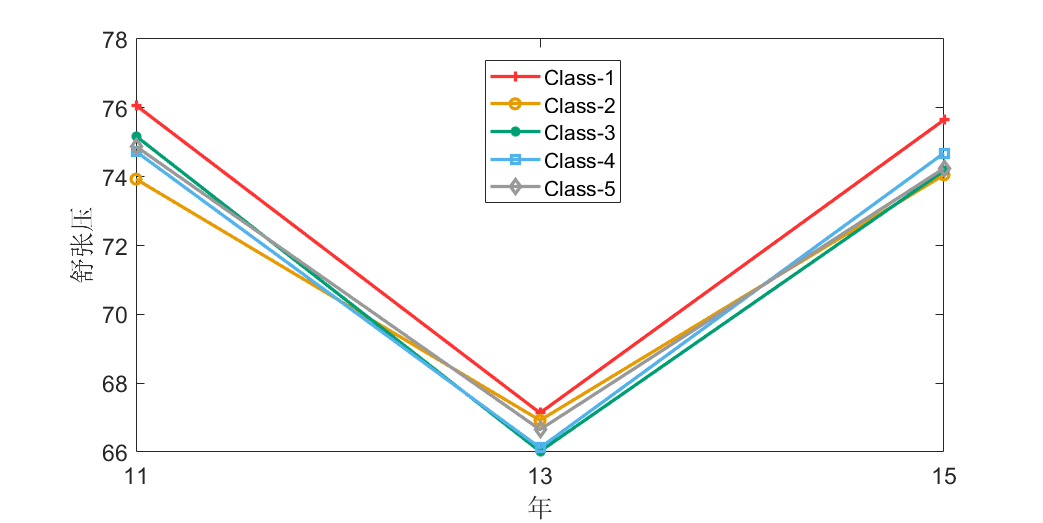


Diastolic pressure


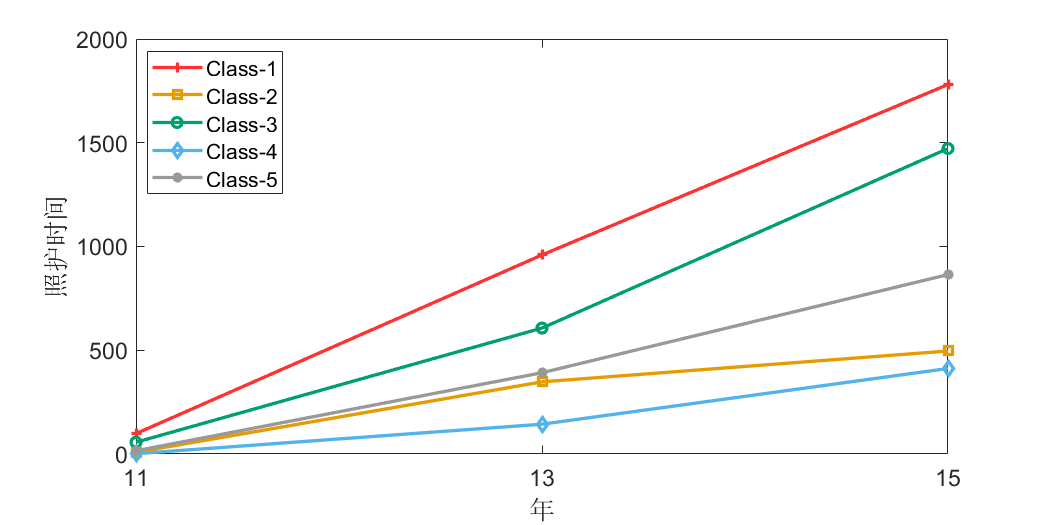


Time of care

## Fig. S8. The distribution of the several major characteristics of the five categories over time (2011 to 2015).

D

C

B

F

E

A

## Fig. S9. The distribution of functional categories in different sex, age and region

*Note.* (A)=Male. (B)=Female. (C)=Less than 70 years old. (D)=70 years of age and older. (E)=Living in urban. (F)=Living in rural. class 1: viability disorder; class 2: acute disease; class 3: somatic functional disorder; class 4: health; class 5: sub-disorder status.


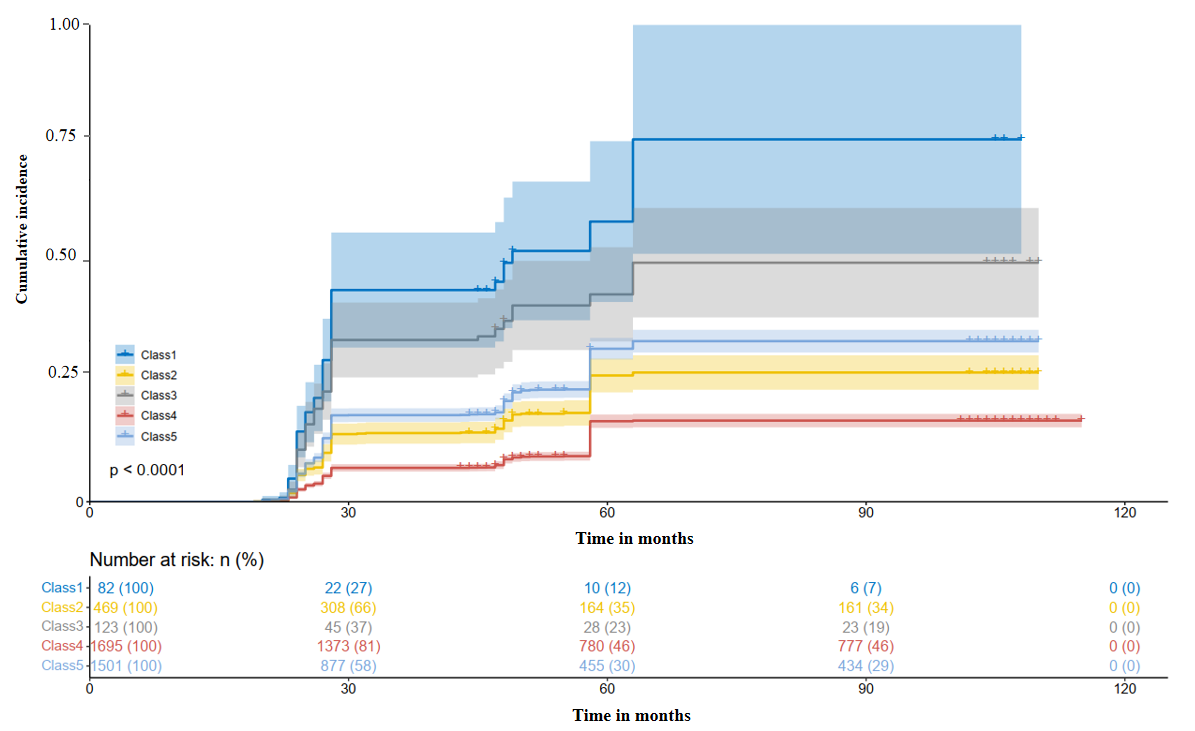


## Fig. S10. The progression of ADL impairment over time by categories

## Table. S1. The selection and assigned values in 29 indicators

| Dimensions-indicators | Assigned values of 29 indicators |
| --- | --- |
| **Self-care** |  |
| ADL | This refers primarily to the ability to maintain basic life care, including the difficulty of bathing, eating, dressing, using the toilet, controlling urine and feces. The measurement of the difficulty degree of each indicator as follows: “with difficulty but able to complete”, “with difficulty and need help”, “unable to complete” =1; “Without any difficulty” =0. |
| IADL | It is the observation variable of the elderly's social activity ability, which consisted of housework, cooking, shopping and financial management, medicine. The measurement of the difficulty degree of each indicator as follows: “with difficulty but able to complete”, “with difficulty and need help”, “unable to complete” =1; “Without any difficulty” =0. |
| Frailty | Physical Frailty Phenotype was used to measure frailty, which consisted of weight loss, weakness, slowness, low energy expenditure, exhaustion. The score range of PFP scale is 0~5. When the score is 3 and higher, it is defined as frailty=1; otherwise=0. |
| **Motor ability** |  |
| Balance | Stand with the heel side of one foot touching the big toe of the other for 10 seconds: “unable to complete” =1; “Without any difficulty” =0. |
| Falls | Have you fallen in the past two years: falls =1; non-falls =0. |
| Postural transition | Get up after sitting in your chair for a long time:“with difficulty but able to complete”, “with difficulty and need help”, “unable to complete”=1; “Without any difficulty” =0. |
| Arm stretching | Extend your arms up along your shoulders: “with difficulty but able to complete”, “with difficulty and need help”, “unable to complete” =1; “Without any difficulty” =0. |
| Mobility | Climb several flights of stairs without stopping:“with difficulty but able to complete”, “with difficulty and need help”, “unable to complete” =1; “Without any difficulty” =0. |
| **Ability of processing diseases** |  |
| Receiving medical services | In the past month, have you visited a medical institution or received in-patient medical care: have been to=1; not have been to=0. |
| Exceptional treatment | Surgical infusion and acupuncture for any treatment: have been to=1; not have been to=0. |
| Self-treatment | In the past month, have you self-treated yourself in any of the following ways (note: prescriptions are not included here)? “buy over-the-counter or prescription drugs”, “use traditional Chinese herbal medicine”, “take health care products such as vitamins”, “use health care equipment” =0; “none” =1. |
| Assistive Device | Do you use the following AIDS: “crutches”, “walkers”, “manual wheelchairs”, “electric wheelchairs” =1; none of them=0. |
| **Cognitive mental status and communication** |  |
| Cognition | The cognitive function of the elderly is usually measured by three aspects of mental state episodic memory ability and visual spatial ability. CHARLS project team asked interviewees to answer five mathematical calculation questions whether they knew the date of the year or month at the time of interview. One point was scored for each correct answer to the questions of the week and season, and the total score was the mental state score (range: 0-10 points). By asking respondents 10 words and asking them to recall the words at two different times, 0.5 points were scored for each pair of words recalled. The mean value of the two recall scores was the score of episodic memory ability (range :0~10 points). Visual spatial ability measured by pressing the Fig. painting, painting for 1 point, otherwise don't score (range: 0 ~ 1). Cognitive function in the elderly by three aspects including mental state, Episodic memory ability and visual spatial ability scored (range: 0 ~ 21 points). When the score was equal to or higher than the average (9.7 points), participants had higher cognitive functions, otherwise had low cognitive function. In this paper, below 9.7 points=1; higher than 9.7=0. |
| Memory | How do you feel about your memory now: “excellent”, “very good”, “good”, “general” =0; “Poor” =1. |
| Vision | Including near object vision and distant vision: “Excellent”, “very good”, “good”, “so-so” =0; “bad” =1. |
| Hearing | How do you feel about your hearing: “Excellent”, “very good”, “good”, “so-so” =0; “bad” =1. |
| Sadness | To feel sad, depressed, or depressed: “No”, “mild”, “moderate” =0; “serious”, “very serious” =1. |
| Depression | The Center for Epidemiologic Studies Depression Scale (CES-D), was used in this paper. CES-D consists of 10 items, in which respondents are asked about their feelings and behaviors in the past week, and are given 0~3 points according to the depression degree reflected in the items from low to high, with a total score of 0~30 points. The higher the score is, the more serious the depression is, and generally 10 points or above are considered as having depressive symptoms. a score of 0~9 was defined as no depressive symptoms=0; a score of 10~30 was defined as having depressive symptoms=1. |
| **Medical condition** |  |
| Chronic disease | Have you been diagnosed with (conditions listed below, read one by one) by a doctor: hypertension, Diabetes, Dyslipidemia, Cancer or malignant tumor, Chronic lung diseases, Liver disease, Heart attack, Stroke, Kidney disease, Stomach or other digestive disease, Emotional, nervous, or psychiatric problems, Memory-related disease, Arthritis or rheumatism, Asthma =1; none=0. |
| Physical disabilities | Whether you have a physical disability: yes=1; none=0. |
| **Sources of care** |  |
| Access of caregiver | If you need to be taken care of in daily life, such as eating, dressing, you will have a family member (other than your spouse) or a friend who can take care of you in the long term: no=1; yes=0. |
| Living arrangement | Living arrangement: “live with their children in a same house”, “in a same dwelling or courtyard” =0; “in a same village”, “in another household in this city” “in another province” =1. |
| **Home settings** |  |
| Elevator in a four-story apartment | Housing types: without elevator=1; with elevator=0. |
| Accessibility | Do you have wheelchair accessible around your place: no=1; yes=0. |
| **Social interactions** |  |
| Interacted with friends | Have you done any of this activity in the last month: no=1; yes=0. |
| Community club | Have you done any of this activity in the last month: no=1; yes=0. |
| Provide help to friends | Have you done any of this activity in the last month: no=1; yes=0. |
| Outdoor activities | Have you done any of this activity in the last month: no=1; yes=0. |
| Community-related organization | Have you done any of this activity in the last month: no=1; yes=0. |

## Table S2. The distribution of functional categories by traditional classification

|  | ADL | IADL | ADL or IADL |
| --- | --- | --- | --- |
| Health | 4567（76.22） | 3598（60.00） | 3499（58.39） |
| Mild disability | 708（11.82） | 1601（26.72） | 1676（27.97） |
| Moderate disability | 469（7.83） | 506（8.44） | 524（8.74） |
| Severe disability | 248（4.14） | 287（4.79） | 293（4.90） |
| Disability rate | 1425（23.78） | 2394（40.00） | 2493（41.61） |

*Note.* ADL or IADL: once any of the ADLs and IADLs has a different degree of functional independence,

the more severe one is selected as the assessment result.

## Table. S3. Conditional probability distributions of multidimensional functional categories

|  | Class1 | Class2 | Class3 | Class4 | Class5 |
| --- | --- | --- | --- | --- | --- |
| ADL | 0.826 | 0.096 | 0.652 | 0.012 | 0.206 |
| IADL | 0.872 | 0.128 | 0.693 | 0.051 | 0.273 |
| Frailty | 0.137 | 0.025 | 0.084 | 0.013 | 0.04 |
| Falls | 0.36 | 0.176 | 0.398 | 0.092 | 0.201 |
| Postural transition | 0.104 | 0.005 | 0.03 | 0 | 0.003 |
| Arm Stretching | 0.44 | 0.051 | 0.359 | 0.013 | 0.106 |
| Balance ability | 0.346 | 0.268 | 0.392 | 0.179 | 0.308 |
| Mobility | 0.599 | 0.083 | 0.421 | 0.01 | 0.143 |
| Assistive Device | 0.445 | 0.038 | 0.226 | 0.013 | 0.052 |
| Self-treatment | 0.613 | 0.544 | 0.635 | 0.394 | 0.558 |
| Receiving medical services | 0.033 | 0.997 | 1 | 0.054 | 0.057 |
| Exceptional treatment | 0 | 0.682 | 0.654 | 0 | 0 |
| Hearing | 0.4 | 0.137 | 0.376 | 0.062 | 0.245 |
| Depression | 0.654 | 0.314 | 0.724 | 0.081 | 0.414 |
| Sadness | 0.039 | 0.015 | 0.048 | 0.001 | 0.016 |
| Memory | 0.627 | 0.324 | 0.701 | 0.139 | 0.531 |
| Cognition | 0.693 | 0.277 | 0.697 | 0.198 | 0.473 |
| Vision | 0.672 | 0.364 | 0.65 | 0.176 | 0.48 |
| Physical Disabilities | 0.51 | 0.151 | 0.42 | 0.083 | 0.255 |
| Chronic disease | 0.904 | 0.839 | 0.936 | 0.589 | 0.788 |
| Access of caregiver | 0.38 | 0.284 | 0.363 | 0.237 | 0.311 |
| Living arrangement | 0.439 | 0.451 | 0.41 | 0.44 | 0.417 |
| Accessibility | 0.724 | 0.73 | 0.732 | 0.786 | 0.733 |
| Elevator in a four-story apartment | 0.984 | 0.961 | 0.995 | 0.922 | 0.981 |
| Interacted with friends | 0.989 | 0.917 | 0.979 | 0.883 | 0.976 |
| Community club | 0.996 | 0.977 | 0.996 | 0.976 | 0.993 |
| Provide help to friends | 0.737 | 0.617 | 0.666 | 0.631 | 0.669 |
| Outdoor activities | 0.933 | 0.806 | 0.926 | 0.775 | 0.86 |
| Community-related organization | 0.981 | 0.958 | 0.956 | 0.942 | 0.959 |

## Table. S4. Analysis of LCA of sex-stratified functional ability

| Events | Male | | | | |  |  | Female | | | |
| --- | --- | --- | --- | --- | --- | --- | --- | --- | --- | --- | --- |
|  | Class 1 | Class 2 | Class 3 | Class 4 | Class 5 |  | Class 1 | Class 2 | Class 3 | Class 4 | Class 5 |
| N (%) | 254(8.5) | 318(10.6) | 174(5.8) | 1257(42) | 991(33.1) |  | 431(14.4) | 300(10) | 289(9.6) | 779(26) | 1199(40) |
| ADL | 0.886 | 0.114 | 0.566 | 0.006 | 0.22 |  | 0.792 | 0.082 | 0.703 | 0.02 | 0.187 |
| IADL | 0.873 | 0.114 | 0.684 | 0.044 | 0.241 |  | 0.87 | 0.138 | 0.709 | 0.063 | 0.294 |
| Frailty | 0.159 | 0.03 | 0.09 | 0.012 | 0.045 |  | 0.119 | 0.021 | 0.081 | 0.015 | 0.034 |
| Falls | 0.309 | 0.14 | 0.287 | 0.08 | 0.2 |  | 0.386 | 0.219 | 0.468 | 0.112 | 0.2 |
| Postural transition | 0.098 | 0.011 | 0.018 | 0 | 0.001 |  | 0.103 | 0 | 0.039 | 0 | 0.004 |
| Arm Stretching | 0.459 | 0.051 | 0.323 | 0.011 | 0.111 |  | 0.422 | 0.055 | 0.379 | 0.015 | 0.101 |
| Balance ability | 0.343 | 0.179 | 0.4 | 0.151 | 0.239 |  | 0.35 | 0.366 | 0.371 | 0.232 | 0.361 |
| Mobility | 0.574 | 0.089 | 0.387 | 0.006 | 0.133 |  | 0.609 | 0.088 | 0.438 | 0.02 | 0.147 |
| Assistive Device | 0.441 | 0.032 | 0.305 | 0.01 | 0.068 |  | 0.44 | 0.044 | 0.177 | 0.016 | 0.037 |
| Self-treatment | 0.646 | 0.56 | 0.652 | 0.379 | 0.591 |  | 0.594 | 0.527 | 0.631 | 0.413 | 0.527 |
| Receiving medical services | 0.056 | 0.995 | 1 | 0.059 | 0.073 |  | 0.034 | 1 | 1 | 0.045 | 0.052 |
| Exceptional treatment | 0 | 0.681 | 0.729 | 0 | 0 |  | 0 | 0.697 | 0.637 | 0 | 0 |
| Hearing | 0.439 | 0.14 | 0.485 | 0.072 | 0.275 |  | 0.372 | 0.121 | 0.316 | 0.036 | 0.226 |
| Depression | 0.589 | 0.241 | 0.693 | 0.068 | 0.371 |  | 0.686 | 0.387 | 0.749 | 0.108 | 0.448 |
| Sadness | 0.025 | 0.02 | 0.026 | 0.001 | 0.011 |  | 0.047 | 0.01 | 0.064 | 0 | 0.021 |
| Memory | 0.615 | 0.283 | 0.695 | 0.121 | 0.502 |  | 0.624 | 0.355 | 0.713 | 0.167 | 0.56 |
| Cognition | 0.582 | 0.17 | 0.616 | 0.139 | 0.321 |  | 0.748 | 0.38 | 0.76 | 0.303 | 0.6 |
| Vision | 0.601 | 0.305 | 0.588 | 0.155 | 0.45 |  | 0.71 | 0.421 | 0.687 | 0.207 | 0.509 |
| Physical Disabilities | 0.556 | 0.166 | 0.465 | 0.093 | 0.308 |  | 0.472 | 0.128 | 0.4 | 0.055 | 0.216 |
| Chronic disease | 0.917 | 0.852 | 0.92 | 0.583 | 0.777 |  | 0.893 | 0.83 | 0.946 | 0.594 | 0.801 |
| Access of caregiver | 0.439 | 0.297 | 0.375 | 0.252 | 0.372 |  | 0.341 | 0.261 | 0.37 | 0.201 | 0.265 |
| Living arrangement | 0.431 | 0.441 | 0.393 | 0.456 | 0.406 |  | 0.444 | 0.47 | 0.418 | 0.418 | 0.425 |
| Accessibility | 0.685 | 0.729 | 0.763 | 0.772 | 0.727 |  | 0.746 | 0.735 | 0.718 | 0.806 | 0.737 |
| Elevator in a four-story apartment | 0.991 | 0.963 | 0.998 | 0.926 | 0.986 |  | 0.98 | 0.955 | 0.995 | 0.918 | 0.976 |
| Interacted with friends | 0.781 | 0.665 | 0.744 | 0.649 | 0.708 |  | 0.709 | 0.553 | 0.626 | 0.598 | 0.641 |
| Community club | 0.896 | 0.762 | 0.906 | 0.749 | 0.803 |  | 0.952 | 0.849 | 0.941 | 0.822 | 0.906 |
| Provide help to friends | 0.991 | 0.953 | 0.96 | 0.944 | 0.946 |  | 0.972 | 0.962 | 0.953 | 0.942 | 0.97 |
| Outdoor activities | 0.988 | 0.936 | 0.966 | 0.893 | 0.977 |  | 0.99 | 0.897 | 0.985 | 0.864 | 0.979 |
| Community-related organization | 0.997 | 0.972 | 1 | 0.975 | 0.996 |  | 0.996 | 0.983 | 0.993 | 0.978 | 0.991 |

## Table. S5. Analysis of LCA of age-stratified functional ability

| Events | Less than 70 | | | | |  | |  | | Older than 70 | | | | |  |
| --- | --- | --- | --- | --- | --- | --- | --- | --- | --- | --- | --- | --- | --- | --- | --- |
|  | Class 1 | Class 2 | Class 3 | Class 4 | Class 5 | |  | | Class 1 | | Class 2 | Class 3 | Class 4 | Class 5 | |
| N (%) | 310(8.1) | 432(11.3) | 255(6.7) | 1456(38.1) | 1367(35.8) | |  | | 375(17.3) | | 186(8.6) | 208(9.6) | 580(26.7) | 823(37.9) | |
| ADL | 0.256 | 0.149 | 0.816 | 0.011 | 0.044 | |  | | 0.852 | | 0.064 | 0.506 | 0.007 | 0.31 | |
| IADL | 0.237 | 0.191 | 0.825 | 0.029 | 0.238 | |  | | 0.966 | | 0.059 | 0.552 | 0.13 | 0.377 | |
| Frailty | 0.015 | 0.018 | 0.094 | 0.007 | 0.027 | |  | | 0.171 | | 0.014 | 0.128 | 0.056 | 0.071 | |
| Falls | 0.232 | 0.19 | 0.397 | 0.094 | 0.116 | |  | | 0.354 | | 0.154 | 0.313 | 0.066 | 0.248 | |
| Postural transition | 0.002 | 0.011 | 0.072 | 0 | 0.002 | |  | | 0.138 | | 0 | 0.028 | 0 | 0.006 | |
| Arm Stretching | 0.146 | 0.083 | 0.435 | 0.013 | 0.044 | |  | | 0.483 | | 0.016 | 0.276 | 0.012 | 0.126 | |
| Balance ability | 0.271 | 0.306 | 0.426 | 0.144 | 0.329 | |  | | 0.291 | | 0.187 | 0.337 | 0.304 | 0.319 | |
| Mobility | 0.133 | 0.122 | 0.477 | 0.005 | 0.079 | |  | | 0.77 | | 0.019 | 0.382 | 0.058 | 0.214 | |
| Assistive Device | 0.033 | 0.018 | 0.295 | 0.01 | 0.011 | |  | | 0.571 | | 0.049 | 0.284 | 0.01 | 0.135 | |
| Self-treatment | 0.719 | 0.516 | 0.634 | 0.408 | 0.335 | |  | | 0.579 | | 0.595 | 0.575 | 0.241 | 0.542 | |
| Receiving medical services | 0.155 | 1 | 0.331 | 0.083 | 0.057 | |  | | 0.075 | | 0.301 | 1 | 0 | 0.073 | |
| Exceptional treatment | 0 | 0.924 | 0.159 | 0 | 0 | |  | | 0 | | 0.139 | 0.856 | 0 | 0 | |
| Hearing | 0.155 | 0.157 | 0.353 | 0.033 | 0.269 | |  | | 0.484 | | 0.073 | 0.378 | 0.195 | 0.314 | |
| Depression | 0.474 | 0.404 | 0.731 | 0.065 | 0.349 | |  | | 0.633 | | 0.129 | 0.621 | 0.103 | 0.398 | |
| Sadness | 0.019 | 0.042 | 0.047 | 0 | 0.018 | |  | | 0.03 | | 0.004 | 0.03 | 0 | 0.009 | |
| Memory | 0.466 | 0.396 | 0.674 | 0.089 | 0.582 | |  | | 0.662 | | 0.123 | 0.636 | 0.319 | 0.543 | |
| Cognition | 0.301 | 0.282 | 0.595 | 0.123 | 0.532 | |  | | 0.871 | | 0.14 | 0.8 | 0.518 | 0.613 | |
| Vision | 0.468 | 0.406 | 0.663 | 0.158 | 0.476 | |  | | 0.716 | | 0.206 | 0.615 | 0.206 | 0.507 | |
| Physical Disabilities | 0.172 | 0.178 | 0.467 | 0.058 | 0.27 | |  | | 0.576 | | 0.1 | 0.398 | 0.187 | 0.331 | |
| Chronic disease | 0.895 | 0.836 | 0.951 | 0.619 | 0.62 | |  | | 0.862 | | 0.789 | 0.903 | 0.395 | 0.795 | |
| Access of caregiver | 0.347 | 0.264 | 0.423 | 0.238 | 0.259 | |  | | 0.333 | | 0.281 | 0.354 | 0.228 | 0.298 | |
| Living arrangement | 0.406 | 0.507 | 0.442 | 0.467 | 0.528 | |  | | 0.425 | | 0.274 | 0.374 | 0.414 | 0.392 | |
| Accessibility | 0.737 | 0.724 | 0.7 | 0.786 | 0.67 | |  | | 0.737 | | 0.851 | 0.769 | 0.748 | 0.757 | |
| Elevator in a four-story apartment | 0.964 | 0.972 | 0.987 | 0.924 | 1 | |  | | 0.996 | | 0.855 | 0.994 | 0.993 | 0.982 | |
| Interacted with friends | 0.615 | 0.62 | 0.747 | 0.617 | 0.779 | |  | | 0.74 | | 0.606 | 0.703 | 0.628 | 0.646 | |
| Community club | 0.804 | 0.812 | 0.937 | 0.759 | 0.943 | |  | | 0.944 | | 0.729 | 0.941 | 0.812 | 0.884 | |
| Provide help to friends | 0.927 | 0.956 | 0.971 | 0.936 | 0.971 | |  | | 0.984 | | 0.945 | 0.984 | 0.972 | 0.98 | |
| Outdoor activities | 0.954 | 0.946 | 0.992 | 0.877 | 1 | |  | | 0.987 | | 0.811 | 0.986 | 0.951 | 0.979 | |
| Community-related organization | 0.991 | 0.987 | 0.997 | 0.978 | 0.994 | |  | | 0.996 | | 0.949 | 1 | 0.994 | 0.991 | |

## Table. S6. Analysis of LCA of region-stratified functional ability

| Events | Rural | | | | |  |  | Urban | | | |
| --- | --- | --- | --- | --- | --- | --- | --- | --- | --- | --- | --- |
|  | Class 1 | Class 2 | Class 3 | Class 4 | Class 5 |  | Class 1 | Class 2 | Class 3 | Class 4 | Class 5 |
| N (%) | 583(12.2) | 481(10.1) | 427(9) | 1443(30.3) | 1832(38.4) |  | 102(8.3) | 137(11.2) | 36(2.9) | 593(48.4) | 358(29.2) |
| ADL | 0.831 | 0.089 | 0.705 | 0.012 | 0.243 |  | 0.804 | 0.131 | 0.04 | 0.003 | 0.16 |
| IADL | 0.878 | 0.137 | 0.734 | 0.065 | 0.298 |  | 0.91 | 0.149 | 0.123 | 0.033 | 0.204 |
| Frailty | 0.156 | 0.04 | 0.075 | 0.019 | 0.042 |  | 0.108 | 0 | 0.02 | 0.004 | 0.021 |
| Falls | 0.359 | 0.164 | 0.434 | 0.09 | 0.218 |  | 0.316 | 0.221 | 0.267 | 0.092 | 0.155 |
| Postural transition | 0.114 | 0.005 | 0.036 | 0 | 0.003 |  | 0.075 | 0.007 | 0 | 0 | 0.003 |
| Arm Stretching | 0.47 | 0.039 | 0.386 | 0.012 | 0.118 |  | 0.345 | 0.103 | 0.057 | 0.009 | 0.103 |
| Balance ability | 0.338 | 0.278 | 0.402 | 0.188 | 0.306 |  | 0.338 | 0.268 | 0.527 | 0.18 | 0.262 |
| Mobility | 0.633 | 0.098 | 0.449 | 0.014 | 0.163 |  | 0.484 | 0.05 | 0.132 | 0.009 | 0.094 |
| Assistive Device | 0.461 | 0.039 | 0.245 | 0.011 | 0.061 |  | 0.413 | 0.047 | 0.042 | 0.015 | 0.037 |
| Self-treatment | 0.587 | 0.508 | 0.638 | 0.364 | 0.567 |  | 0.704 | 0.62 | 0.574 | 0.426 | 0.661 |
| Receiving medical services | 0.013 | 1 | 1 | 0.051 | 0.089 |  | 0.192 | 1 | 0.209 | 0.077 | 0 |
| Exceptional treatment | 0 | 0.808 | 0.653 | 0 | 0 |  | 0.089 | 0.487 | 0.116 | 0 | 0.003 |
| Hearing | 0.427 | 0.149 | 0.375 | 0.082 | 0.252 |  | 0.302 | 0.082 | 1 | 0.061 | 0.039 |
| Depression | 0.67 | 0.341 | 0.737 | 0.1 | 0.445 |  | 0.589 | 0.28 | 0.472 | 0.051 | 0.314 |
| Sadness | 0.034 | 0.024 | 0.049 | 0.001 | 0.02 |  | 0.054 | 0 | 0 | 0.002 | 0.006 |
| Memory | 0.64 | 0.367 | 0.694 | 0.195 | 0.547 |  | 0.589 | 0.252 | 0.732 | 0.075 | 0.352 |
| Cognition | 0.742 | 0.347 | 0.707 | 0.281 | 0.489 |  | 0.549 | 0.109 | 0.551 | 0.055 | 0.277 |
| Vision | 0.682 | 0.384 | 0.664 | 0.198 | 0.489 |  | 0.635 | 0.346 | 0.646 | 0.154 | 0.409 |
| Physical Disabilities | 0.513 | 0.155 | 0.441 | 0.112 | 0.263 |  | 0.513 | 0.136 | 0.525 | 0.049 | 0.126 |
| Chronic disease | 0.894 | 0.818 | 0.944 | 0.557 | 0.791 |  | 0.939 | 0.917 | 0.897 | 0.646 | 0.881 |
| Access of caregiver | 0.358 | 0.246 | 0.391 | 0.196 | 0.31 |  | 0.43 | 0.414 | 0.285 | 0.331 | 0.378 |
| Living arrangement | 0.446 | 0.456 | 0.397 | 0.466 | 0.417 |  | 0.453 | 0.444 | 0.434 | 0.386 | 0.364 |
| Accessibility | 0.707 | 0.723 | 0.737 | 0.741 | 0.726 |  | 0.806 | 0.748 | 0.773 | 0.881 | 0.798 |
| Elevator in a four-story apartment | 1 | 1 | 0.998 | 0.997 | 0.996 |  | 0.888 | 0.822 | 0.965 | 0.741 | 0.868 |
| Interacted with friends | 0.732 | 0.609 | 0.661 | 0.638 | 0.65 |  | 0.815 | 0.645 | 0.71 | 0.636 | 0.713 |
| Community club | 0.948 | 0.825 | 0.924 | 0.803 | 0.859 |  | 0.88 | 0.77 | 0.835 | 0.725 | 0.842 |
| Provide help to friends | 0.984 | 0.966 | 0.954 | 0.941 | 0.95 |  | 0.993 | 0.947 | 1 | 0.951 | 0.979 |
| Outdoor activities | 0.993 | 0.974 | 0.985 | 0.972 | 0.992 |  | 0.951 | 0.713 | 0.876 | 0.664 | 0.866 |
| Community-related organization | 0.995 | 0.993 | 0.995 | 0.987 | 0.993 |  | 1 | 0.94 | 1 |  | 0.98 |

## Table. S7. Participants characteristics among 5 categories

|  | Class1 | Class2 | Class3 | Class4 | Class5 | P value |
| --- | --- | --- | --- | --- | --- | --- |
| N | 685（11.4） | 618（10.3） | 463（7.7） | 2036（34.0） | 2190（36.6） |  |
| Sex |  |  |  |  |  |  |
| Male | 254（37.0） | 318（51.5） | 174（37.6） | 1257（61.8） | 991（45.2） | <0.001 |
| Female | 431（63.0） | 300（48.5） | 289（62.4） | 779（38.2） | 1199（54.8） |  |
| Age | 71.54±7.63 | 67.04±5.98 | 69.36±7.25 | 66.76±5.88 | 68.25±6.64 |  |
| Marital status，% |  |  |  |  |  |  |
| Married | 463（67.6） | 506（81.9） | 345（74.5） | 1712（84.1） | 1679（35.7） | <0.001 |
| Divorced | 217（31.7） | 111（18.0） | 116（25.1） | 305（15.0） | 489（22.3） |  |
| unmarried | 5（0.7） | 1（0.2） | 2（0.4） | 19（38.8） | 22（1.0） |  |
| Educational level，% |  |  |  |  |  |  |
| illiteracy | 387（56.5） | 185（29.9） | 248（53.6） | 469（23.0） | 923（42.1） | <0.001 |
| Primary school | 239（34.9） | 302（48.9） | 188（40.6） | 992（48.7） | 1000（45.7） |  |
| High school | 54（7.9） | 117（18.9） | 26（5.6） | 507（24.9） | 246（11.2） |  |
| Bachelor and above | 5（0.7） | 14（2.3） | 1（0.2） | 68（3.4） | 21（1.0） |  |
| Place of residence |  |  |  |  |  |  |
| Rural | 583（85.1） | 481（77.8） | 427（92.2） | 1443（70.9） | 1832（83.7） |  |
| Urban | 102（14.9） | 137（10.3） | 36（7.8） | 593（29.1） | 358（16.3） |  |
| Numbers of children，% |  |  |  |  |  |  |
| 0 | 177（25.8） | 158（25.6） | 122（26.3） | 572（28.1） | 633（28.9） | <0.001 |
| 1~3 | 168（24.5） | 203（32.8） | 106（22.9） | 753（37.0） | 639（29.2） |  |
| 4~5 | 220（32.1） | 192（31.3） | 156（33.7） | 515（25.3） | 629（28.7） |  |
| >5 | 120（17.5） | 65（10.5） | 79（17.1） | 196（9.6） | 289（13.2） |  |
| BMI | 22.60$\pm$4.50 | 22.91$\pm$3.71 | 22.74±4.41 | 23.04±3.89 | 22.84±4.23 | <0.001 |
| Systolic pressure | 141.56.09±30.50 | 132.15±24.93 | 136.49±23.85 | 134.98±24.78 | 136.30±25.04 |  |
| Diastolic pressure | 76.73±12.70 | 74.01±12.33 | 75.39±12.77 | 75.25±11.20 | 75.00±12.02 |  |
| Sleeping duration | 5.60±2.41 | 6.07±3.47 | 5.33±2.39 | 6.63±1.64 | 6.09±2.07 |  |
| Hours of informal care | 33.73±92.07 | 2.15±19.17 | 16.09±52.7 | 0.49±8.17 | 4.79±29.99 | <0.001 |
| Hours of formal care | 83.55±143.61 | 6.95±48.15 | 47.80±95.17 | 1.33±14.18 | 14.85±59.44 | <0.001 |
| Satisfaction |  |  |  |  |  |  |
| Extremely | 8（1.2） | 8（1.3） | 3（0.6） | 53（2.6） | 27（0.5） | <0.001 |
| Very much | 97（14.2） | 126（20.4） | 61（13.2） | 497（24.4） | 427（19.5） |  |
| Relative | 430（62.8） | 419（67.8） | 296（63.9） | 1346（66.1） | 1449（66.2） |  |
| A little | 114（16.6） | 58（9.4） | 74（16.0） | 130（6.4） | 237（10.8） |  |
| Not at all | 36（5.3） | 7（1.1） | 29（6.3） | 10（0.5） | 50（2.3） |  |
| Self-rated health |  |  |  |  |  | <0.001 |
| Very good | 4（0.6） | 10（1.6） | 2（0.4） | 145（7.1） | 45（2.1） |  |
| Good | 25（3.6） | 52（8.4） | 13（2.8） | 455（22.3） | 205（9.4） |  |
| Fair | 174（25.4） | 264（42.7） | 110（23.8） | 1072（52.7） | 1055（48.2） |  |
| Bad | 317（46.3） | 235（38.0） | 224（48.4） | 338（16.6） | 717（32.7） |  |
| Very bad | 165（24.1） | 57（9.3） | 114（24.6） | 26（1.3） | 168（7.7） |  |

## Table. S8. Univariate Logistic Regression Analysis of mortality presence based on baseline Data

| Indicators/variables | B | Wald | P | OR | Lower limits | Upper limits |
| --- | --- | --- | --- | --- | --- | --- |
| Classification | | 84.702 | 0 |  |  |  |
| Class1 vs Class5 | 1.297 | 48.202 | 0 | 3.658 | 2.537 | 5.276 |
| Class2 vs Class5 | 0.378 | 2.351 | 0.125 | 1.459 | 0.9 | 2.366 |
| Class3 vs Class5 | 1.083 | 24.272 | 0 | 2.954 | 1.92 | 4.544 |
| Class4 vs Class5 | -0.226 | 1.255 | 0.263 | 0.798 | 0.537 | 1.184 |
| Place of residence, Rural vs Urban | 0.311 | 2.837 | 0.092 | 1.365 | 0.95 | 1.961 |
| Educational level | | 13.451 | 0.004 |  |  |  |
| Illiteracy vs bachelor and above | 1.684 | 2.781 | 0.095 | 5.386 | 0.745 | 38.957 |
| Primary school vs bachelor and above | 1.353 | 1.796 | 0.18 | 3.871 | 0.535 | 28.028 |
| High school vs bachelor and above | 0.976 | 0.905 | 0.342 | 2.655 | 0.355 | 19.855 |
| Marital status | | 35.761 | 0 |  |  |  |
| Married vs single | -0.328 | 0.203 | 0.652 | 0.721 | 0.173 | 2.997 |
| Divorced vs single | 0.524 | 0.514 | 0.473 | 1.689 | 0.403 | 7.074 |
| Sex, male Vs Female | 0.252 | 3.388 | 0.066 | 1.286 | 0.984 | 1.682 |
| Age | 0.107 | 146.125 | 0 | 1.113 | 1.093 | 1.132 |
| Household per capita income, yuan | 0 | 0.009 | 0.923 | 1 | 1 | 1 |
| Number of children | | 28.085 | 0 |  |  |  |
| 0 vs 6-7 | -0.692 | 12.847 | 0 | 0.5 | 0.343 | 0.731 |
| 1-3 vs 6-7 | -1.04 | 26.198 | 0 | 0.354 | 0.237 | 0.526 |
| 4-5 vs 6-7 | -0.706 | 13.47 | 0 | 0.493 | 0.338 | 0.72 |
| BMI | -0.086 | 25.067 | 0 | 0.918 | 0.887 | 0.949 |
| ADL, yes vs no | 0.929 | 45.228 | 0 | 2.533 | 1.932 | 3.321 |
| IADL, yes vs no | 0.921 | 45.504 | 0 | 2.512 | 1.922 | 3.283 |
| Frailty, yes vs no | 1.243 | 35.139 | 0 | 3.468 | 2.299 | 5.231 |
| Falling, yes vs no | 0.166 | 1.024 | 0.312 | 1.18 | 0.856 | 1.627 |
| Postural transition | | 46.797 | 0 |  |  |  |
| Inability to complete vs achieveable | 1.787 | 38.503 | 0 | 5.974 | 3.397 | 10.507 |
| With difficulty and need help vs achieveable | 0.982 | 11.941 | 0.001 | 2.669 | 1.529 | 4.657 |
| With difficulty but achieveable vs achieveable | 0.19 | 1.533 | 0.216 | 1.21 | 0.895 | 1.635 |
| Arm Stretching, no vs yes | 0.641 | 14.628 | 0 | 1.898 | 1.367 | 2.636 |
| Balance ability, no vs yes | -0.002 | 0 | 0.989 | 0.998 | 0.739 | 1.346 |
| Mobility |  | 87.974 | 0 |  |  |  |
| Inability to complete vs achieveable | 1.419 | 75.659 | 0 | 4.131 | 3.001 | 5.687 |
| With difficulty and need help vs achieveable | 0.595 | 3.48 | 0.062 | 1.813 | 0.97 | 3.386 |
| With difficulty but achieveable vs achieveable | 0.207 | 1.227 | 0.268 | 1.23 | 0.853 | 1.773 |
| Assistive Device, yes vs no | 1.62 | 117.71 | 0 | 5.052 | 3.77 | 6.769 |
| Self-treatment, yes vs no | 0.178 | 1.719 | 0.19 | 1.195 | 0.915 | 1.561 |
| Receiving medical services, yes vs no | 0.418 | 7.767 | 0.005 | 1.519 | 1.132 | 2.037 |
| Exceptional treatment, yes vs no | 0.511 | 8.139 | 0.004 | 1.667 | 1.174 | 2.369 |
| Hearing |  | 15.882 | 0.003 |  |  |  |
| Poor vs Excellent | 1.157 | 1.295 | 0.255 | 3.181 | 0.433 | 23.349 |
| Fair vs Excellent | 0.729 | 0.516 | 0.472 | 2.073 | 0.284 | 15.141 |
| Good vs Excellent | 0.656 | 0.415 | 0.519 | 1.928 | 0.262 | 14.197 |
| Very good vs Excellent | 0.068 | 0.004 | 0.948 | 1.071 | 0.136 | 8.45 |
| Depression, yes vs no | 0.342 | 6.15 | 0.013 | 1.407 | 1.074 | 1.844 |
| Sadness |  | 0.64 | 0.726 |  |  |  |
| Poor vs good | 0.359 | 0.594 | 0.441 | 1.432 | 0.575 | 3.57 |
| Fair vs good | 0.045 | 0.076 | 0.783 | 1.046 | 0.76 | 1.439 |
| Memory |  | 2.856 | 0.582 |  |  |  |
| Poor vs Excellent | -0.364 | 0.124 | 0.725 | 0.695 | 0.092 | 5.276 |
| Fair vs Excellent | -0.527 | 0.259 | 0.611 | 0.59 | 0.078 | 4.487 |
| Good vs Excellent | -0.206 | 0.039 | 0.844 | 0.814 | 0.105 | 6.298 |
| Very good vs Excellent | -0.439 | 0.162 | 0.687 | 0.645 | 0.076 | 5.461 |
| Cognition, bad vs good | 0.92 | 43.134 | 0 | 2.51 | 1.907 | 3.303 |
| Vision, bad vs good | 0.235 | 2.954 | 0.086 | 1.264 | 0.968 | 1.652 |
| Physical Disabilities, yes vs no | 0.454 | 9.562 | 0.002 | 1.575 | 1.181 | 2.1 |
| Pain, yes vs no | 0.042 | 0.09 | 0.764 | 1.043 | 0.791 | 1.377 |
| Chronic disease, yes vs no | 0.535 | 8.618 | 0.003 | 1.708 | 1.195 | 2.442 |
| Access of caregiver, no vs yes | -0.126 | 0.679 | 0.41 | 0.882 | 0.654 | 1.189 |
| Living arrangement | | 8.595 | 0.072 |  |  |  |
| A same dwelling or courtyard vs a same house | 0.273 | 0.278 | 0.598 | 1.314 | 0.476 | 3.624 |
| A same village vs a same house | -0.299 | 0.293 | 0.588 | 0.742 | 0.251 | 2.187 |
| Another household in this city vs a same house | 0.108 | 0.041 | 0.84 | 1.114 | 0.39 | 3.182 |
| Another province vs a same house | 0.404 | 0.592 | 0.442 | 1.498 | 0.535 | 4.192 |
| Accessibility, no vs yes | 0.086 | 0.29 | 0.59 | 1.09 | 0.797 | 1.492 |
| Elevator in a four-story apartment, no vs yes | 0.835 | 2.693 | 0.101 | 2.305 | 0.85 | 6.251 |
| Outdoor activities, no vs yes | 0.855 | 4.194 | 0.041 | 2.351 | 1.037 | 5.329 |
| Community-related organization, no vs yes | 17.973 | 0 | 0.997 | 63925787 | 0 | . |
| Interacted with friends, no vs yes | 0.14 | 0.915 | 0.339 | 1.151 | 0.863 | 1.534 |
| Community club, no vs yes | 0.223 | 1.251 | 0.263 | 1.25 | 0.846 | 1.847 |
| Provide help to friends, no vs yes | 0.963 | 3.585 | 0.058 | 2.619 | 0.967 | 7.095 |

## Table S9. Univariate Logistic Regression Analysis of ADLs impairment presence based on baseline Data.

| Indicators/variables | B | Wald | *P* | OR | 95% CI | |
| --- | --- | --- | --- | --- | --- | --- |
|  |  |  |  |  | Lower limits | Upper limits |
| **Place of residence, Rural vs Urban** | 0.166 | 4.128 | 0.042 | 1.18 | 1.006 | 1.385 |
| **Falling, yes vs no** | 0.455 | 26.337 | 0 | 1.575 | 1.324 | 1.874 |
| **IADL, yes vs no** | 0.641 | 55.73 | 0 | 1.898 | 1.604 | 2.245 |
| **Frailty, yes vs no** | 0.774 | 14.508 | 0 | 2.168 | 1.456 | 3.227 |
| **Postural transition** |  | 140.47 | 0 |  |  |  |
| Inability to complete vs Achieveable | 1.444 | 4.794 | 0.029 | 4.236 | 1.163 | 15.423 |
| With difficulty and need help vs Achieveable | 1.829 | 27.471 | 0 | 6.227 | 3.142 | 12.339 |
| With difficulty but achieveable vs Achieveable | 0.89 | 114.292 | 0 | 2.435 | 2.068 | 2.866 |
| **Arm Stretching, no vs yes** | 0.812 | 35.183 | 0 | 2.253 | 1.723 | 2.947 |
| **Balance ability, no vs yes** | 0.29 | 15.247 | 0 | 1.337 | 1.155 | 1.546 |
| **Mobility** |  | 185.373 | 0 |  |  |  |
| Inability to complete vs Achieveable | 1.181 | 101.27 | 0 | 3.258 | 2.588 | 4.1 |
| With difficulty and need help vs Achieveable | 1.373 | 47.293 | 0 | 3.948 | 2.67 | 5.84 |
| With difficulty but achieveable vs Achieveable | 0.707 | 86.532 | 0 | 2.027 | 1.747 | 2.353 |
| **Assistive Device, yes vs no** | 1.275 | 42.491 | 0 | 3.579 | 2.439 | 5.252 |
| **Self-treatment, yes vs no** | 0.381 | 34.701 | 0 | 1.463 | 1.289 | 1.661 |
| **Receiving medical services, yes vs no** | 0.301 | 13.604 | 0 | 1.351 | 1.151 | 1.585 |
| **Exceptional treatment, yes vs no** | 0.412 | 14.628 | 0 | 1.51 | 1.222 | 1.864 |
| **Hearing** |  | 55.638 | 0 |  |  |  |
| Poor vs Excellent | 0.332 | 1.063 | 0.303 | 1.394 | 0.741 | 2.621 |
| Fair vs Excellent | -0.042 | 0.017 | 0.895 | 0.959 | 0.516 | 1.783 |
| Good vs Excellent | -0.224 | 0.493 | 0.482 | 0.8 | 0.428 | 1.492 |
| Very good vs Excellent | -0.551 | 2.814 | 0.093 | 0.577 | 0.303 | 1.097 |
| **Depression, yes vs no** | 0.772 | 108.513 | 0 | 2.163 | 1.871 | 2.501 |
| **Sadness** |  | 10.591 | 0.005 |  |  |  |
| Poor vs good | 1.024 | 10.512 | 0.001 | 2.785 | 1.499 | 5.173 |
| Fair vs good | 0.036 | 0.209 | 0.647 | 1.036 | 0.889 | 1.208 |
| **Memory** |  | 71.968 | 0 |  |  |  |
| Poor vs Excellent | 0.305 | 0.185 | 0.667 | 1.356 | 0.338 | 5.445 |
| Fair vs Excellent | -0.203 | 0.082 | 0.775 | 0.816 | 0.204 | 3.275 |
| Good vs Excellent | -0.175 | 0.061 | 0.805 | 0.839 | 0.208 | 3.39 |
| Very good vs Excellent | -0.742 | 1.04 | 0.308 | 0.476 | 0.114 | 1.982 |
| **Cognition, bad vs good** | 0.551 | 65.712 | 0 | 1.736 | 1.519 | 1.983 |
| **Vision, bad vs good** | 0.556 | 67.467 | 0 | 1.743 | 1.527 | 1.99 |
| **Physical Disabilities, yes vs no** | 0.533 | 40.831 | 0 | 1.704 | 1.447 | 2.007 |
| **Chronic disease, yes vs no** | 0.492 | 47.091 | 0 | 1.635 | 1.421 | 1.881 |
| **Pain, yes vs no** | 0.827 | 125.974 | 0 | 2.286 | 1.979 | 2.641 |
| **Access of caregiver, no vs yes** | 0.113 | 2.385 | 0.123 | 1.119 | 0.97 | 1.292 |
| **Living arrangement** |  | 16.781 | 0.002 |  |  |  |
| A same dwelling or courtyard vs a same house | 0.413 | 2.978 | 0.084 | 1.512 | 0.945 | 2.418 |
| A same village vs a same house | 0.237 | 0.907 | 0.341 | 1.268 | 0.778 | 2.067 |
| Another household in this city vs a same house | 0.282 | 1.307 | 0.253 | 1.326 | 0.817 | 2.152 |
| Another province vs a same house | 0.594 | 5.886 | 0.015 | 1.811 | 1.121 | 2.926 |
| **Accessibility, no vs yes** | 0.04 | 0.303 | 0.582 | 1.041 | 0.902 | 1.203 |
| **Elevator in a four-story apartment, no vs yes** | 0.326 | 3.481 | 0.062 | 1.385 | 0.984 | 1.951 |
| **Interacted with friends, no vs yes** | -0.041 | 0.366 | 0.545 | 0.96 | 0.842 | 1.095 |
| **Community club, no vs yes** | 0.145 | 2.907 | 0.088 | 1.156 | 0.979 | 1.366 |
| **Provide help to friends, no vs yes** | 0.391 | 6.321 | 0.012 | 1.479 | 1.09 | 2.007 |
| **Outdoor activities, no vs yes** | 0.21 | 2.506 | 0.113 | 1.234 | 0.951 | 1.6 |
| **Community-related organization, no vs yes** | 0.171 | 0.429 | 0.513 | 1.187 | 0.711 | 1.981 |
| **Marital status** |  | 32.905 | 0 |  |  |  |
| Married vs single | 0.169 | 0.195 | 0.659 | 1.185 | 0.559 | 2.512 |
| Divorced vs single | 0.65 | 2.784 | 0.095 | 1.915 | 0.893 | 4.109 |
| **Sex, Male Vs Female** | 0.428 | 43.825 | 0 | 1.535 | 1.352 | 1.742 |
| Age | 0.055 | 101.82 | 0 | 1.056 | 1.045 | 1.068 |
| **Number of children** |  | 31.149 | 0 |  |  |  |
| 0 vs 6-7 | -0.191 | 2.809 | 0.094 | 0.826 | 0.661 | 1.033 |
| 1-3 vs 6-7 | -0.492 | 19.172 | 0 | 0.612 | 0.491 | 0.762 |
| 4-5 vs 6-7 | -0.104 | 0.836 | 0.36 | 0.901 | 0.722 | 1.126 |
| **Household per capita income, yuan** | 0 | 1.815 | 0.178 | 1 | 1 | 1 |
| **BMI** | 0.016 | 4.032 | 0.045 | 1.016 | 1 | 1.032 |
| **Educational level** |  | 45.614 | 0 |  |  |  |
| Illiteracy vs bachelor and above | 1.117 | 12.117 | 0 | 3.056 | 1.629 | 5.734 |
| Primary school vs bachelor and above | 0.862 | 7.278 | 0.007 | 2.369 | 1.266 | 4.433 |
| High school vs bachelor and above | 0.533 | 2.678 | 0.102 | 1.705 | 0.9 | 3.23 |
| **Classification** |  | 252.498 | 0 |  |  |  |
| Class1 vs Class5 | 1.371 | 18.72 | 0 | 3.939 | 2.117 | 7.329 |
| Class2 vs Class5 | -0.329 | 9.557 | 0.002 | 0.72 | 0.584 | 0.887 |
| Class3 vs Class5 | 0.739 | 11.646 | 0.001 | 2.093 | 1.369 | 3.199 |
| Class4 vs Class5 | -0.987 | 182.161 | 0 | 0.373 | 0.323 | 0.43 |

## Table S10. Cox regression analysis comparing mortality and ADLs impairments among categories

|  |  | **Events (%)** | **HR (95%CI)** | **P** |
| --- | --- | --- | --- | --- |
| Mortality |  |  |  |  |
|  | Class1 | 152(22.2) | 1 | -- |
|  | Class2 | 59(9.5) | 0.408 (0.302-0.551) | <0.001 |
|  | Class3 | 93(20.1) | 0.898 (0.694-1.162) | 0.414 |
|  | Class4 | 159(7.8) | 0.329 (0.263-0.411) | <0.001 |
|  | Class5 | 234(10.7) | 0.453 (0.370-0.556) | <0.001 |
| ADLs impairments |  |  |  |  |
|  | Class1 | 70(85.4) | 1 | -- |
|  | Class2 | 242(51.6) | 0.523 (0.394-0.696) | <0.001 |
|  | Class3 | 93(75.6) | 0.868 (0.632-1.192) | 0.380 |
|  | Class4 | 603 (35.6) | 0.326 (0.249-0.428) | <0.001 |
|  | Class5 | 896(49.2) | 0.597 (0.461-0.772) | <0.001 |

*Note*. class 1: viability disorders; class 2: acute disease; class 3: somatic functional disorders;

class 4: health; class 5: sub-disorder status

## Table S11. Competitive risk modeling of the incidence of ADLs impairments in different categories

| Factor | SHR | STD | Z | *P* | 95%*CI* | |
| --- | --- | --- | --- | --- | --- | --- |
| **Sex, male vs Female** | 1.099 | 0.120 | 0.870 | 0.385 | 0.888 | 1.361 |
| **Age** | 1.032 | 0.008 | 4.250 | 0.000 | 1.017 | 1.048 |
| **Marital status** |  |  |  |  |  |  |
| Married vs single | 1.335 | 0.173 | 2.240 | 0.025 | 1.036 | 1.720 |
| Divorces vs single | 0.572 | 0.359 | -0.890 | 0.373 | 0.167 | 1.956 |
| **BMI** | 1.021 | 0.010 | 2.110 | 0.035 | 1.001 | 1.042 |
| **Place of residence, Rural vs Urban** | 1.456 | 0.192 | 2.850 | 0.004 | 1.125 | 1.885 |
| **Educational level** | |  |  |  |  |  |
| Illiteracy vs bachelor and above | 1.464 | 0.171 | 3.260 | 0.001 | 1.165 | 1.841 |
| Primary school vs bachelor and above | 0.894 | 0.167 | -0.600 | 0.550 | 0.620 | 1.289 |
| High school vs bachelor and above | 0.852 | 0.523 | -0.260 | 0.794 | 0.255 | 2.840 |
| **Classification** | |  |  |  |  |  |
| Class 2 vs Class 1 | 0.536 | 0.134 | -2.480 | 0.013 | 0.328 | 0.877 |
| Class 3 vs Class 1 | 0.981 | 0.264 | -0.070 | 0.943 | 0.579 | 1.661 |
| Class 4 vs Class 1 | 0.268 | 0.064 | -5.510 | 0.000 | 0.168 | 0.428 |
| Class 5 vs Class 1 | 0.514 | 0.112 | -3.050 | 0.002 | 0.335 | 0.789 |
| **Number of children** | |  |  |  |  |  |
| 0 vs 6-7 | 0.707 | 0.094 | -2.610 | 0.009 | 0.545 | 0.917 |
| 1-3 vs 6-7 | 0.807 | 0.104 | -1.660 | 0.098 | 0.627 | 1.040 |
| 4-5 vs 6-7 | 0.662 | 0.115 | -2.370 | 0.018 | 0.471 | 0.931 |
| **sleep duration** | 0.953 | 0.024 | -1.930 | 0.053 | 0.908 | 1.001 |
